# Modulation of microRNA editing, expression and processing by ADAR2 deaminase in glioblastoma

**DOI:** 10.1186/s13059-014-0575-z

**Published:** 2015-01-13

**Authors:** Sara Tomaselli, Federica Galeano, Shahar Alon, Susanna Raho, Silvia Galardi, Vinicia Assunta Polito, Carlo Presutti, Sara Vincenti, Eli Eisenberg, Franco Locatelli, Angela Gallo

**Affiliations:** Department of Pediatric Oncohaematology, RNA Editing Laboratory, Bambino Gesù Children’s Hospital IRCCS, Piazza S. Onofrio 4, Rome, 00165 Italy; Department of Neurobiology, George S. Wise Faculty of Life Sciences, Tel-Aviv University, Tel-Aviv, 69978 Israel; Department of Biomedicine and Prevention, University of Rome Tor Vergata, Via Montpellier 1, Rome, 00133 Italy; Department of Genetics and Molecular Biology, Sapienza University of Rome, Via Dei Sardi 70, Rome, 00100 Italy; Raymond and Beverly Sackler School of Physics and Astronomy, Tel-Aviv University, Tel-Aviv, 69978 Israel; Department of Pediatric Science, Università di Pavia, Strada Nuova 65, Pavia, 27100 Italy

## Abstract

**Background:**

ADAR enzymes convert adenosines to inosines within double-stranded RNAs, including microRNA (miRNA) precursors, with important consequences on miRNA retargeting and expression. ADAR2 activity is impaired in glioblastoma and its rescue has anti-tumoral effects. However, how ADAR2 activity may impact the miRNome and the progression of glioblastoma is not known.

**Results:**

By integrating deep-sequencing and array approaches with bioinformatics analyses and molecular studies, we show that ADAR2 is essential to edit a small number of mature miRNAs and to significantly modulate the expression of about 90 miRNAs in glioblastoma cells. Specifically, the rescue of ADAR2 activity in cancer cells recovers the edited miRNA population lost in glioblastoma cell lines and tissues, and rebalances expression of onco-miRNAs and tumor suppressor miRNAs to the levels observed in normal human brain. We report that the major effect of ADAR2 is to reduce the expression of a large number of miRNAs, most of which act as onco-miRNAs. ADAR2 can edit miR-222/221 and miR-21 precursors and decrease the expression of the corresponding mature onco-miRNAs *in vivo* and *in vitro*, with important effects on cell proliferation and migration.

**Conclusions:**

Our findings disclose an additional layer of complexity in miRNome regulation and provide information to better understand the impact of ADAR2 editing enzyme in glioblastoma. We propose that ADAR2 is a key factor for maintaining edited-miRNA population and balancing the expression of several essential miRNAs involved in cancer.

**Electronic supplementary material:**

The online version of this article (doi:10.1186/s13059-014-0575-z) contains supplementary material, which is available to authorized users.

## Background

MicroRNAs (miRNAs or miRs) are small non-coding single-stranded RNAs that play a crucial role in many cellular pathways by silencing RNA targets by either inhibiting their translation or promoting their degradation [1]. miRNAs are transcribed in the nucleus and the nascent transcripts are called primary miRNAs (pri-miRNAs). Pri-miRNAs can be several kilobases long and contain one or more secondary stem-loop structures [2]. The nuclear pri-miRNA transcripts are cleaved by a nuclear RNAse III enzyme, Drosha [2], which acts in concert with several co-factors, including DGCR8 [3], to generate stem-loop precursor miRNAs (pre-miRNAs). Pre-miRNAs undergo a second cleavage by the cytoplasmic RNAse III enzyme Dicer, which, in cooperation with other cofactors, cuts the loop end of pre-miRNAs. The resulting product is an approximately 22 nucleotide RNA duplex composed of the mature miRNA guide and a passenger strand (also referred to as miRNA*). Once loaded into the RNA-induced silencing complex (RISC), the mature miRNA strand is able to recognize the target RNA through a six to eight nucleotide seed region at the 5′ end of the miRNA, while the passenger strand is usually degraded [4].

miRNAs are important regulatory RNAs involved in numerous cellular processes, including proliferation, differentiation and development. Since miRNAs can act as oncogenes or tumor suppressors [5], fluctuations of their expression are important factors in both normal and pathological conditions, including cancer [1,5]. Recently, miRNAs have been shown to be differentially expressed in malignant astrocytomas and glioblastomas (also known as astrocytoma grade IV) compared with normal brain, with some miRNAs, such as miR-21, miR-221 and miR-222, being particularly over-expressed in cancers [6,7].

Adenosine (A) to inosine (I) RNA editing, which is mediated by the ADARs, adenosine deaminase enzymes acting on double-stranded RNAs (dsRNAs), is a widespread post-transcriptional mechanism in mammals that affects several coding and regulatory RNAs, including miRNA precursors, by altering their sequence and structure. Indeed, inosines are recognized as guanosines (G) by the splicing and translation machineries and dsRNAs containing I**∙**U wobble base pairs can interfere with the action of RNA-binding proteins such as Dicer [8]. Therefore, A-to-I RNA editing events, equivalent to A-to-G cDNA changes, may induce amino acid substitutions, alter RNA splicing sites and perturb dsRNA structures. In mammals, there are three ADAR enzymes: ADAR1 and ADAR2 are active deaminases, whereas ADAR3 seems to be an inactive enzyme [9]. All ADARs have a similar domain structure with a catalytic domain at the carboxyl terminus and two or three dsRNA-binding domains at the amino terminus [9]. Recently, it has been shown that ADARs can interact with the miRNA world in both an editing-dependent and -independent way [10,11]. Specifically, ADARs can edit miRNA precursors and thus alter their maturation steps [12]. Moreover, when editing occurs within the miRNA seed, this can lead to redirection of the edited miRNA to a different subset of mRNA targets [13]. It has also been shown that ADARs can impair miRNA maturation independently of their catalytic domains, with ADAR1 able to globally enhance miRNA biogenesis by directly interacting with Dicer [14,15].

ADAR2 is an essential enzyme for brain development and function [16,17]. In previous studies, we and others showed that ADAR2 activity is impaired in glioblastoma in both children and adults [18-20] and that the decrease of ADAR2-mediated editing correlates with increased tumor grade in children [18]. Moreover, we recently demonstrated that ADAR2 deaminase activity is sufficient to inhibit glioblastoma proliferation and tumor growth, as it modulates the CDC14B/Skp2/p21/p27 pathway in adult glioblastoma cell lines and this was further confirmed in different grades of pediatric glioma [21].

Due to the importance of ADAR2 activity in glioblastoma and the link between ADARs and miRNAs, we decided to analyze miRNA profiles in glioblastoma cells upon ADAR2 over-expression or silencing as well as in normal brain and glioblastoma tissues, with the aim of identifying important ADAR2-regulated miRNAs in glioblastoma. We found that ADAR2 specifically edits a small number of mature miRNAs. Moreover, it is able to modulate the expression of many miRNAs, most of which are involved in tumorigenesis. In particular, we found that the maturation steps of the important oncogenic miR-221, -222 and -21 are inhibited by ADAR2 editing activity, with consequent effects on the proliferative and migratory capacities of glioblastoma cells.

## Results

### ADAR2 editing activity alters the miRNA expression profile in glioblastoma

A-to-I RNA editing within miRNA precursors can alter their maturation steps and consequently the expression levels of mature miRNAs [12]. To investigate the effects mediated by ADAR2 editing activity on the miRNome (microRNA expression and editing profile), we used both microarray and deep-sequencing approaches, followed by an extensive bioinformatic analysis, in human brain and glioblastoma tissues as well as in a glioblastoma cell line (U118) in which we modulated ADAR2 expression and/or activity.

Microarray expression analysis was performed to compare RNA isolated from glioblastoma U118 cells transfected with either the active ADAR2 or the inactive ADAR2 enzyme (ADAR2 E/A) [21]. ADAR2 E/A carries a single point mutation (E/A) in the catalytic domain, which renders it unable to promote the deamination reaction but still able to bind dsRNAs [22]. Both the active and inactive ADAR2 were expressed at similar levels in the stably transfected U118 cell lines (Figure S1 in Additional file 1). Three independent miRNA-array experiments were performed using total RNA extracted from U118 over-expressing either ADAR2 or ADAR2 E/A. In these cells, exogenous ADAR2 is expressed at approximately three-fold the level of the endogenous protein levels and this is sufficient to rescue the normal editing levels at well-known editing sites [21]. We selected only miRNAs exhibiting a log2(ratio) of at least ±0.5 (corresponding to ≥1.41 relative fold-change) (Additional file 2, ADAR2 versus ADAR2 E/A, E column). For the statistical analysis we applied the standard Benjamini-Hochberg multiple testing correction adopting a cutoff false discovery rate (FDR) ≤0.2 (Additional file 2; see Materials and methods). Over-expression of the active ADAR2 caused significant changes in the expression of several miRNAs (46 miRNAs), compared with the inactive ADAR2 E/A (Additional file 2). Among the down-regulated miRNAs, miR-29b, miR-221 and miR-21 were the most reduced in ADAR2 cells (-1.82, -1.65 and -1.59 log-fold, respectively; Additional file 2, column in green). Strikingly, 40 out of 46 of the ADAR2 target miRNAs are involved in cancer development/progression (Additional file 2, column in light brown). Specifically, approximately 80% (13/19) of the down-regulated miRNAs and approximately 30% (8/27) of the over-expressed miRNAs play a role in glioblastoma (Additional file 2, column in light gray). Of note, approximately 52% (10/19) of miRNAs down-regulated by ADAR2 are usually over-expressed and act as onco-miRNAs in glioblastoma (Additional file 2, column in dark gray). Moreover, approximately 20% (5/27) of miRNAs up-regulated by ADAR2 have a role as tumor suppressors and/or are usually down-regulated in glioblastoma (Additional file 2, column in dark gray).

No significant changes in miRNA expression were detected between ADAR2 E/A transfected cells and the control U118 cell line (Additional file 2, untransfected versus ADAR2 E/A).

In summary, miRNA-array data showed that ADAR2 activity restricts the expression of several well-known onco-miRNAs in glioblastoma (Additional file 2, ADAR2 versus ADAR2 E/A, column in dark gray), with mir-221, miR-21, miR-125b and miR-222 being the most down-regulated.

### ADAR2 edits a few miRNAs and alters the expression of many miRNAs

Current miRNA arrays have pre-designed probe sequences that do not give information about the possible sequence modification events within miRNAs. Additionally, any editing events within the mature sequence could alter the binding between the probe and the miRNAs. Therefore, we also carried out a deep-sequencing analysis of small RNA fractions isolated from the U118, U118 ADAR2, U118 ADAR2 E/A and U118 siADAR2 cells (in which we silenced the over-expressed ADAR2 enzyme) [21]. Additionally, we further extended our analysis to healthy human brain and glioblastoma tissues.

Mature miRNAs from these samples were sequenced using the Illumina HiSeq2000 platform. No significant A-to-G changes were identified in the U118 untransfected cells (Table 1), confirming the notion that ADAR2 is either inactive or has greatly reduced activity in high-grade astrocytoma/glioblastoma cell lines [18,23]. Introducing the active ADAR2 into U118 cells, we identified 19 editing sites within 18 miRNAs (Table 1). Nine sites were also found to be edited at statistically significant levels in human brains whereas only two of these were edited in glioblastoma tissue (Table 1). We are confident that these miRNAs are specifically edited by ADAR2, since (i) the inactive ADAR2 E/A cell line shows no significant A-to-G changes and (ii) decreased editing values were observed in the ADAR2-silenced cell line (siADAR2 versus ADAR2 cells) (Table 1). Most of these edited miRNAs had already been identified in our previous study [23]; however, here we detected new potential editing sites in miR-210, miR-503 and miR-3157* (shown in italics in Table 1). The identified editing sites were characterized by an enrichment of uridine (U) in the upstream nucleotide position, with guanosine (G) usually as the downstream nucleotide and with the nucleotide opposing the editing site usually being a cytidine (C) or a uridine (U). These sequence patterns are consistent with genuine A-to-I editing [24]. Of note, 9 out of 19 of the editing sites identified in the ADAR2 transfected cells (Table 1) are located within the miRNA seed region (including the new miR-503). Importantly, editing within the seed sequence may lead to miRNA retargeting [13].

**Table 1:** **ADAR2-mediated editing events in brain and glioblastoma cells and tissues**

**Chr**	**Strand**	**miRNA**	**U118**	**ADAR2**	**ADAR2 E/A**	**siADAR2**	***P*** **-value**	**Pooled human brain**	**Human frontal lobe**	**Glioblastoma**	**Location in pre-miR**	**Location in mature miR**
Chr9	+	hsa-let-7d*	0.3	**2.0**	0	0.5	1.61E-10	**1.2**	**0.7**	0	66	5
Chr17	**-**	**hsa-mir-22**	0	**0.1**	0	0.1	5.50E-34	0	0	0	67	15
Chr19	**-**	hsa-mir-24–2*	0.1	**16.4**	0	**7.1**	<1E-155	0.8	2.3	ND	18	6
Chr19	**-**	hsa-mir-27a*	0.1	**22.5**	0.1	**9.8**	<1E-155	ND	0	**2.6**	10	1
Chr21	**+**	hsa-mir-99a	0.1	**13.8**	0	**6.9**	<1E-155	**5.0**	**1.2**	0	13	1
Chr11	**-**	hsa-mir-100	0	**0.2**	0	**0.1**	1.22E-145	0	0	0	13	1
Chr3	**+**	hsa-mir-138–1*	0	**0.8**	0	**0.5**	2.57E-35	2.1	0.2	ND	74	12
Chr8	**-**	hsa-mir-151	0.1	**0.3**	0	0.2	5.70E-13	0.9	**0.6**	ND	49	3
Chr11	**-**	*hsa-mir-210*	0	**0.5**	0	**0.5**	2.31E-11	ND	ND	0	77	12
Chr14	**+**	hsa-mir-411	0	**12.6**	0	**5.6**	4.31E-39	**15.3**	**13.9**	**3.2**	20	5
Chrx	**-**	hsa-mir-421	0.2	**13.8**	0.5	**9.6**	<1E-155	**1.8**	**1.0**	0	61	14
Chr9	**+**	**hsa-mir-455**	0	**14.2**	0	**5.7**	<1E-155	**1.2**	0.9	0	32	17
Chr17	**-**	hsa-mir-497	0	**26.1**	0	**16.7**	1.73E-155	**6.2**	0.6	0	25	2
Chrx	**-**	***hsa-mir-503***	0	**5.9**	0	1.7	8.75E-14	ND	ND	0	7	2
Chr7	**-**	hsa-mir-589*	2.3	**9.5**	2.2	2.9	1.36E-31	**70.0**	**74.1**	0	66	6
Chr19	**-**	hsa-mir-641	0	**17.0**	0	**8.8**	5.83E-116	0	0	0	17	2
Chr19	**-**	hsa-mir-641	0	**3.0**	0	0.9	2.45E-14	0	**3.6**	0	18	3
Chr10	**-**	hsa-mir-3157*	0	**69.4**	0	**54.5**	1.55E-90	ND	ND	0	70	13
Chr16	**+**	hsa-mir-3176	0	**10.9**	0	**8.2**	6.73E-41	ND	ND	0	74	15

A-to-G changes in mature miRNAs isolated from U118 cell lines (untreated, ADAR2, ADAR2 E/A, siADAR2), human brains (pooled and frontal lobe) and glioblastoma. The statistically significant modifications are marked in bold; the edited miRNAs that were not identified in our previous analysis are indicated in italics [23]; miRNAs that are both edited and modulated by ADAR2 are indicated in bold (Additional files 2 and 3).

Editing levels are represented as percentages. ND, not determined (with <10 reads).

Next, we extended our analysis to possible alterations of miRNA expression mediated by ADAR2 in glioblastoma cells and in human brain tissues (both normal brain and glioblastoma), detecting differences in expression that cannot be explained by the expected Poisson noise [25]. We first analyzed the modulation of miRNA expression in ADAR2 versus ADAR2 E/A U118 cells and in siADAR2 versus ADAR2 U118 cells, focusing our attention on those miRNAs that were up- or down-regulated by ADAR2 and which reversed their expression-trend in the siADAR2 cells (Additional file 3). We selected mature miRNAs with (i) normalized counts greater than 200 reads (ADAR2 plus ADAR2 E/A reads) and (ii) a log2(ratio) between ADAR2 and ADAR2 E/A exceeding 0.5 (in absolute value) (Additional file 3). A total of 91 miRNAs with statistically significant differences in expression were identified (Additional file 3), with 60 miRNAs down- and 31 up-regulated miRNAs in ADAR2 versus ADAR2 E/A cells (Additional file 3), indicating that ADAR2 preferentially restricts miRNA expression. This trend was also observed when we concentrated on the most highly expressed miRNAs (ADAR2 plus ADAR2 E/A ≥10.000 reads): 13 out of 16 miRNAs were down-regulated and only 3 out of 16 miRNAs were up-regulated by ADAR2 (Table 2). Of note, almost all the miRNAs significantly modulated by ADAR2 were also known to be involved in cancer development/progression (Table 2).

**Table 2:** **miRNAs highly expressed in glioblastoma cells and modulated by ADAR2**

**Chr**	**miRNA**	**ADAR2**	**ADAR2 E/A**	**siADAR2**	**log2(ratio) of ADAR2 versus ADAR2 E/A**	**log2(ratio) of siADAR2 versus ADAR2**	**Involvement in cancer**
Chr9	hsa-let-7d	0	18,813	13,703	−14.1995	13.7423	Yes
Chr2	hsa-miR-10b	0	11,101	559	−13.4385	9.1293	Yes
Chr7	hsa-miR-335*	5,296	14,463	5,463	−1.4492	0.0448	No
Chr5	hsa-miR-143	61,575	159,453	81,440	−1.3727	0.4034	Yes
Chr7	hsa-miR-25	29,424	58,238	35,063	−0.9849	0.2529	Yes
Chr19	hsa-let-7e	30,846	56,890	36,482	−0.8831	0.2421	Yes
Chr7	hsa-miR-29a	33,734	59,096	49,119	−0.8088	0.5421	Yes
Chr17	hsa-miR-21	919,239	1,506,954	1,384,694	−0.7131	0.1581	Yes
Chr17	hsa-miR-21*	53,970	84,276	106,392	−0.6430	0.9791	Yes
Chr3	hsa-miR-138-1*	4,460	6,714	5,063	−0.5900	0.1829	Yes
Chrx	hsa-miR-222	277,271	410,972	348,219	−0.5677	0.3287	Yes
Chr19	hsa-miR-769	6,339	9,225	6,433	−0.5412	0.0212	No
Chr6	hsa-miR-30a*	9,650	13,716	13,235	−0.5072	0.4619	Yes
Chr10	hsa-miR-146b	12,692	6,639	10,948	0.9348	−0.2132	Yes
Chr2	hsa-miR-548 s	11,559	8	22	10.3269	−8.3083	No
Chr19	hsa-miR-520c	14,093	0	0	13.7828	−13.7828	Yes

Highly expressed mature miRNAs in U118 glioblastoma cells (ADAR2 plus ADAR2 E/A ≥10,000 reads).

miRNA expression was also analyzed in human healthy brain and glioblastoma tissues, as glioblastoma tissues have reduced ADAR2 activity [18-20]. We selected mature miRNAs with (i) normalized counts greater than 200 reads and (ii) a log2(ratio) between human brain and glioblastoma exceeding 0.5 (in absolute value) (Additional file 4). We identified a total of 293 differently expressed miRNAs between brain and glioblastoma (Additional file 4). Of these miRNAs, 56 were among those significantly expressed in U118 cells and modulated by ADAR2. ADAR2 ‘corrected’ about 50% of the miRNAs (27/56) that were deregulated in glioblastoma (shown in gray in Additional file 4).

Comparing miRNA editing (Table 1) and expression (Additional file 2 and Additional file 3), we observed that relatively few miRNAs were both edited and significantly modulated by ADAR2 (shown in bold in Table 1): miR-22, miR-503 (Additional file 2), miR-138-1* and miR-455 (Additional file 3).

Overall, we identified four miRNAs that are both edited and differentially expressed by ADAR2 (shown in bold in Table 1), 14 miRNAs that are edited but not significantly modulated by ADAR2 (Table 1) and 89 miRNAs that are modulated (either up-regulated or down-regulated) by ADAR2 but not edited within their mature sequence (Additional file 3; excluding the edited miR-138-1* and miR-455).

### ADAR2 activity reduces the levels of the oncogenic miR-221, -222 and -21 by blocking maturation of their precursors

ADAR2 editing activity is progressively lost in astrocytomas from low to high grade in children [18]. Interestingly, we observed that the rescue of active ADAR2 in glioblastoma cells (U118) results in the down-regulation of a large number of miRNAs (Additional file 3), including three important onco-miRNAs (miR-221, miR-222 and miR-21), which play a pivotal role in cancer progression and are found particularly over-expressed in glioblastoma [26,27].

**Figure 1:**
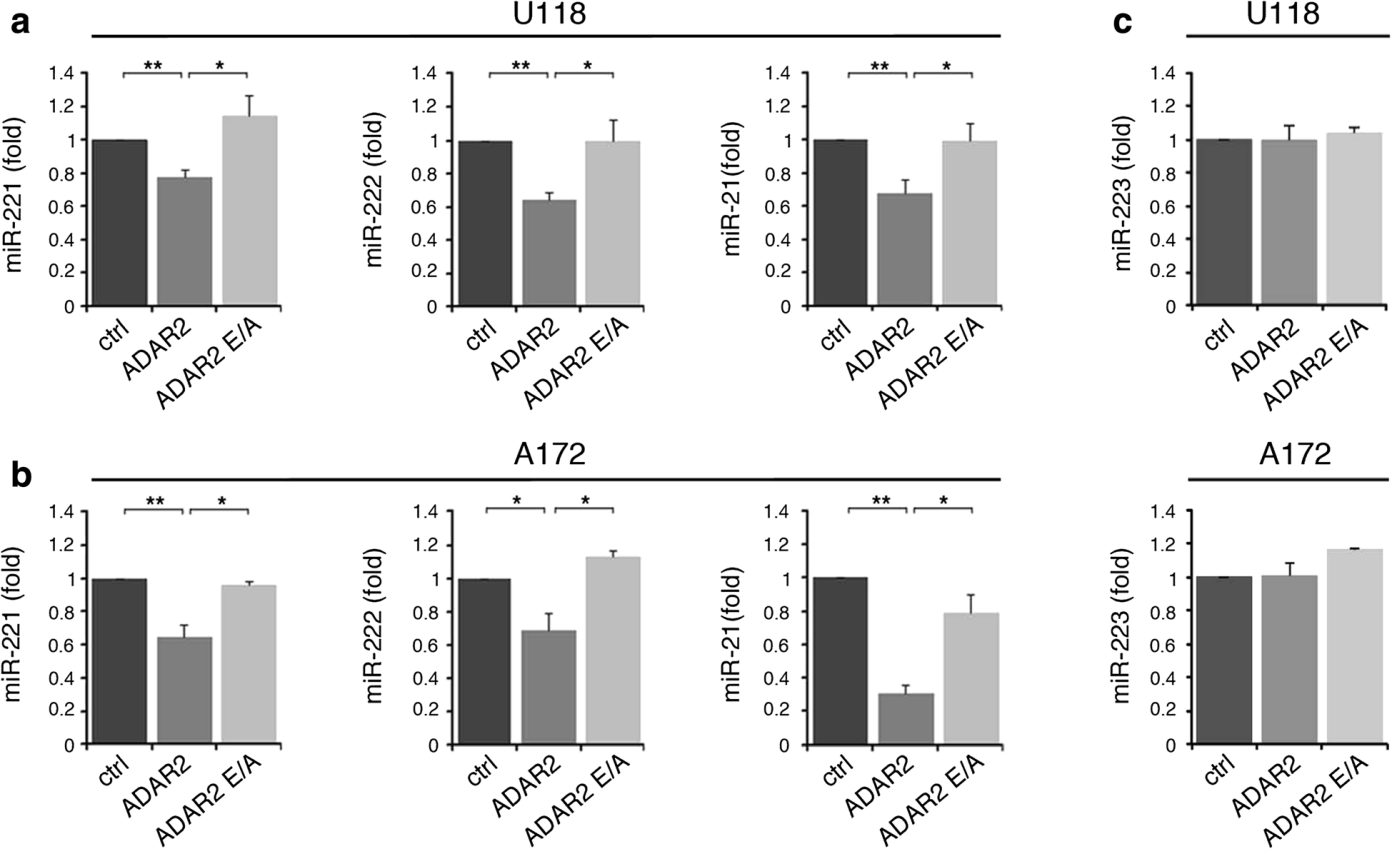
**Down-regulation of selected onco-miRNAs (miR-221, miR-222 and miR-21) in glioblastoma cell lines upon ADAR2 expression. (a)** Mature miR-221, miR-222 and miR-21 expression levels are shown in untreated (ctrl, dark gray), ADAR2 over-expressed (ADAR2, medium gray) and inactive ADAR2 over-expressed (ADAR2 E/A, light gray) U118 cell lines. Values represent the mean of at least three independent quantitative RT-PCRs (qRT-PCRs). Error bars represent standard error of the mean (s.e.m.) (n = 3), **P < 0.01, *P < 0.05. **(b)** The same experiments shown in (a) were performed in the A172 cell lines. **(c)** Mature miR-223 expression levels are shown in U118 (upper panel) and in A172 (lower panel) cell lines. Mean ± s.e.m. (n = 2). All the samples were normalized to RNU6B levels. The expression levels were calculated as a relative-fold increase compared with untreated cells (ctrl) arbitrarily set to 1.

In order to validate the deep-sequencing and microarray data and to explore the connection between ADAR2 activity and miR-221, miR-222 and miR-21, we used two glioblastoma cell lines (U118 and A172) stably over-expressing either ADAR2 or its inactive form ADAR2 E/A at similar levels (Figure S1 in Additional file 1 and data not shown). By using these cell lines, we confirmed that the active ADAR2 editing enzyme significantly decreases the levels of mature miR-221, miR-222 and miR-21 compared with the controls (untreated and ADAR2 E/A cells), as tested by quantitative real-time PCR (qRT-PCR) (Figure 1a, b) and northern blotting analysis (Figure S2 in Additional file 1). As miRNA control, we analyzed the levels of miR-223 (Figure 1c), which were found to be unchanged by ADAR2 as observed in miRNA array and deep-sequencing experiments.

**Figure 2:**
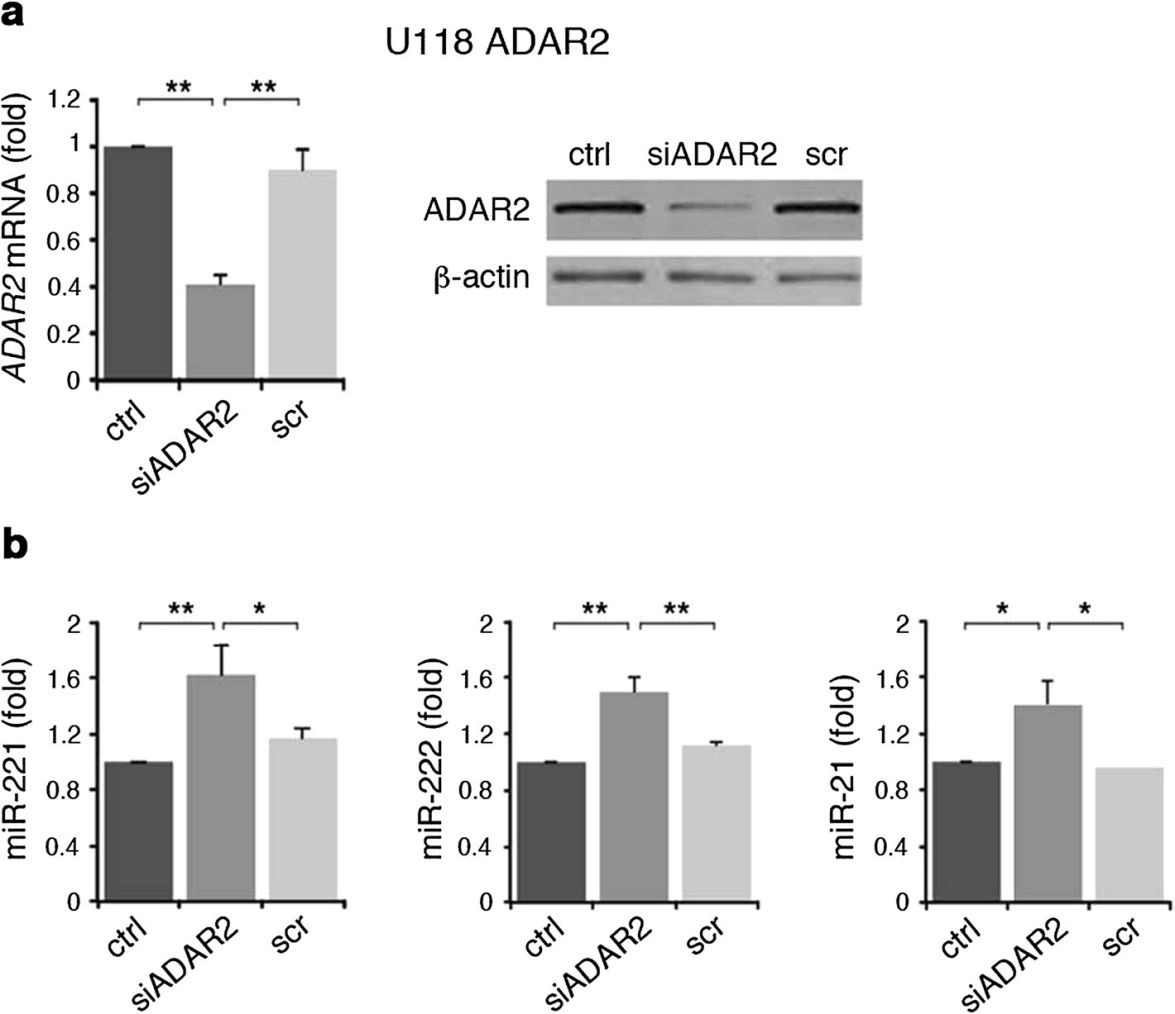
**ADAR2 silencing in U118 cell line increases miR-221, miR-222 and miR-21 expression levels. (a)** Left: qRT-PCRs of *ADAR2* in U118 ADAR2 (ctrl, dark gray) and the same cell line stably transfected with siADAR2 (siADAR2, medium gray) or scramble (scr, light gray) plasmids. Each sample was normalized to *GAPDH* mRNA levels. Mean ± standard error of the mean (s.e.m.) (n = 3), **P < 0.01. Right: a representative immunoblot of ADAR2 in the cell lines analyzed. **(b)** Mature miR-221 (left panel), miR-222 (middle panel) and miR-21 (right panel) expression levels in U118 ADAR2, siADAR2 and scramble cell lines are shown. Each sample was normalized to RNU6B levels. Mean ± s.e.m. (n = 3), **P < 0.01, *P < 0.05. The expression levels were calculated as a relative-fold increase compared with U118 ADAR2 (ctrl) arbitrarily set to 1.

Conversely, silencing of ADAR2 in U118 ADAR2 cells (Figure 2a) increased the expression levels of mature miR-221, miR-222 and miR-21 (Figure 2b). Similar results were also found in A172 silenced cells (data not shown).

**Figure 3:**
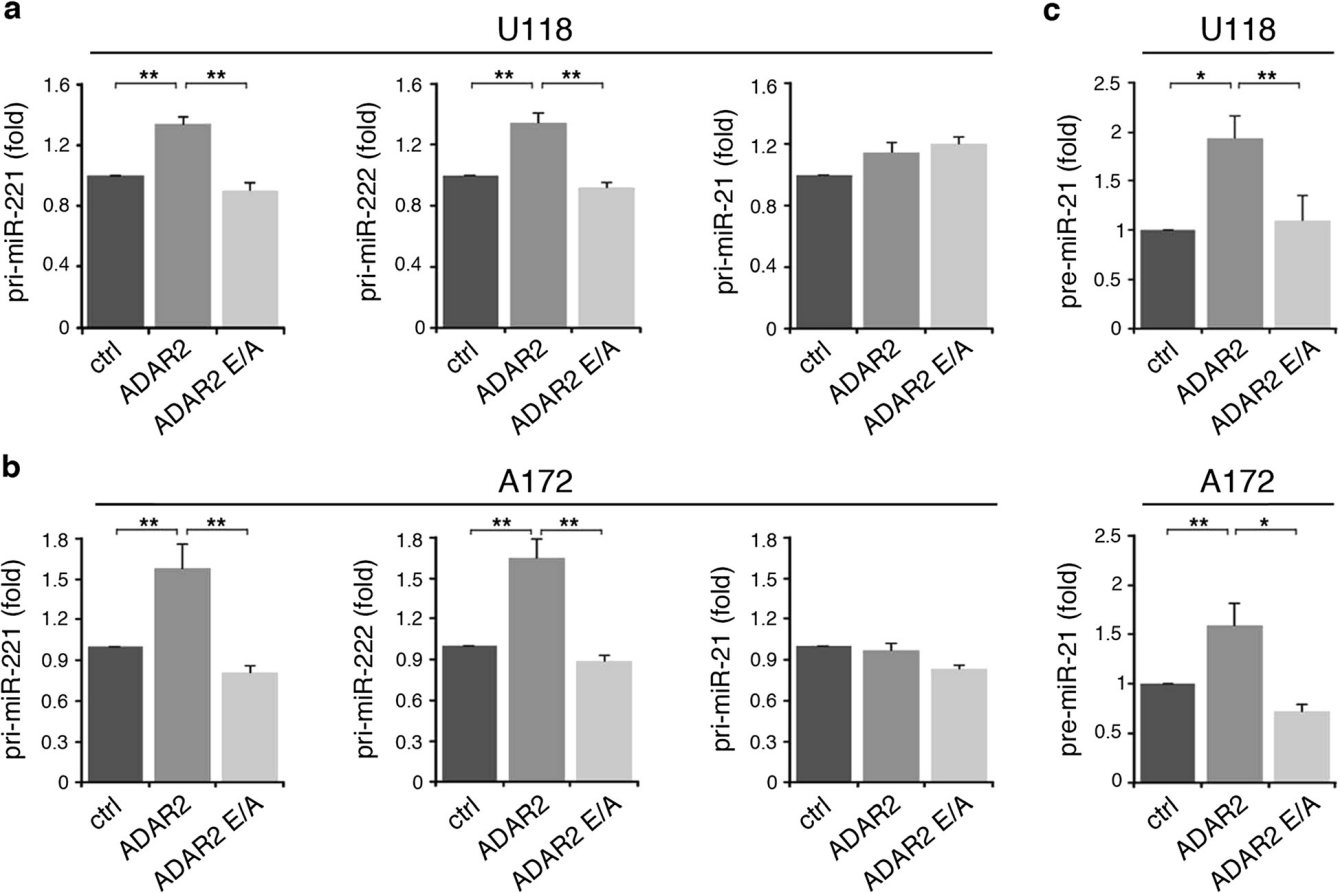
**Modulation of miR-221, miR-222 and miR-21 precursors in glioblastoma cell lines upon ADAR2 and ADAR2 E/A expression. (a)** Pri-miR-221, pri-miR-222 and pri-miR-21 expression levels analyzed by qRT-PCR in U118 untreated (ctrl, dark gray), U118 over-expressing ADAR2 (ADAR2, medium gray) and U118 over-expressing ADAR2 E/A (ADAR2 E/A, light gray) cell lines. Each sample was normalized to *GAPDH* mRNA levels. Mean ± standard error of the mean (s.e.m.) (n = 3), **P < 0.01. **(b)** The same set of experiments in (a) was performed in the A172 cell line. **(c)** Pre-miR-21 expression levels in U118 (upper panel) and A172 (bottom panel) cell lines were analyzed by qRT-PCR. For the pre-miRs assay each sample was normalized to RNU6B levels. Mean ± s.e.m. (n = 3), **P < 0.01, *P < 0.05. The expression levels were calculated as a relative-fold increase compared with untreated cells (ctrl) arbitrarily set to 1.

It has been shown that ADARs can alter the structure and the sequence of miRNA precursors, thus blocking Drosha and Dicer activity, resulting in reduced mature miRNAs and in concomitant accumulation of their precursors [11,28]. Therefore, we tested the precursor levels of miR-221, miR-222 and miR-21 in the ADAR2-modified cell lines. While the levels of mature miR-221, miR-222 and miR-21 decreased (Figure 1a, b), the corresponding precursors (pri- or pre-miRNAs) accumulated in both U118 and A172 cells upon ADAR2 expression (Figure 3). Specifically, we observed that the endogenous pri-miR-221 and pri-miR-222 levels increase in ADAR2 glioblastoma cells compared with both the inactive ADAR2 E/A and the untreated cells (Figure 3a, b). In the case of miR-21 it was the pre-miR-21 rather than the pri-miR-21 that accumulated in the ADAR2 glioblastoma cells, as tested by qRT-PCR (Figure 3c) and northern blotting analysis (Figure S3 in Additional file 1).

Overall, these data demonstrate that ADAR2 deaminase activity results in accumulation of miR-222/221 and miR-21 precursors in two glioblastoma cell lines, with a concomitant reduction of the corresponding mature onco-miRNAs.

### Adar2 inhibits miR-221, miR-222 and miR-21 expression *in vivo*

**Figure 4:**
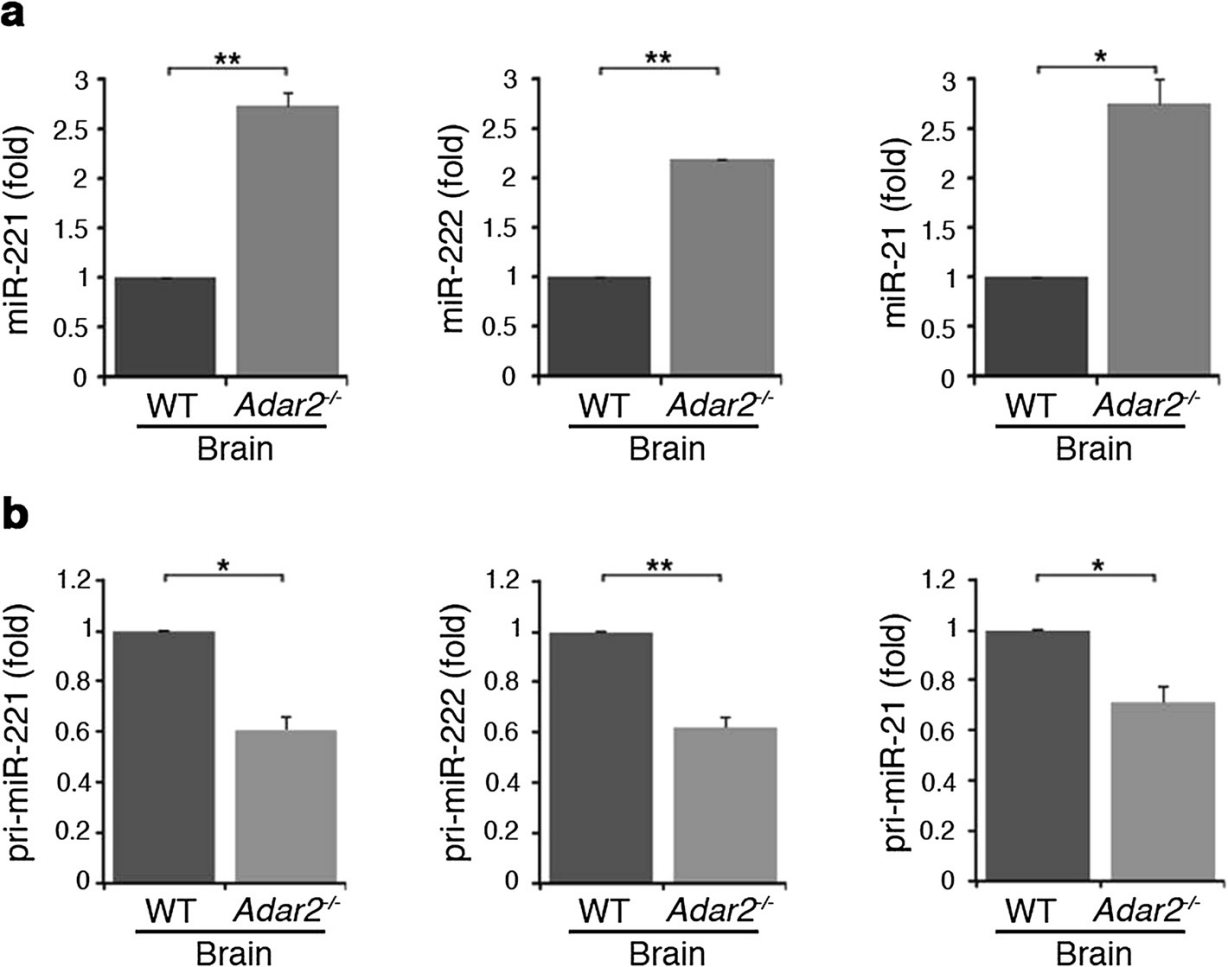
**Altered expression of mature and primary miR-221, -222 and -21 in wild-type and** ***Adar2*** ^**-/-**^ **mouse brain tissue. (a)** Mature miR-221 (left panel), miR-222 (middle panel) and miR-21 (right panel) expression levels were analyzed using specific qRT-PCRs in wild-type (WT, dark gray) and *Adar2* ^-/-^ (medium gray) mouse brain tissue. **(b)** Primary miR-221 (left panel), pri-miR-222 (middle panel) and pri-miR-21 (right panel) expression levels were measured using specific qRT-PCRs in the same samples. Mature miRNAs were normalized to RNU6B and pri-miRNAs were normalized to β-actin levels. The expression levels were calculated as a relative-fold increase compared with the wild-type samples arbitrarily set to 1. Mean ± standard error of the mean (n = 3), **P < 0.01, *P < 0.05.

In order to verify our observation *in vivo*, we took advantage of RNA samples extracted from *Adar2*^*-/-*^ and wild-type mouse brains. If Adar2 is important for the maturation of miR-221, miR-222 and miR-21 in physiological conditions, we would expect a substantial increase in expression of these three miRNAs in the absence of *Adar2*. We examined the endogenous levels of miR-221, miR-222 and miR-21 and of their precursors by qRT-PCR and found that their mature miRNA are indeed significantly over-expressed in the *Adar2*^*-/-*^ mouse brain (+2.7-, +2.2- and +2.7-fold, respectively) compared with the wild type (Figure 4a). We also observed an approximately 40% decrease in the level of their precursors (pri-miRs) in the absence of *Adar2* (Figure 4b).

Our results indicate that the ADAR2 enzyme controls miRNA expression, not only in cancer cells, but also in normal mammalian brain.

### ADAR2 edits the pri-miR-221, -222 and -21 precursors

**Figure 5:**
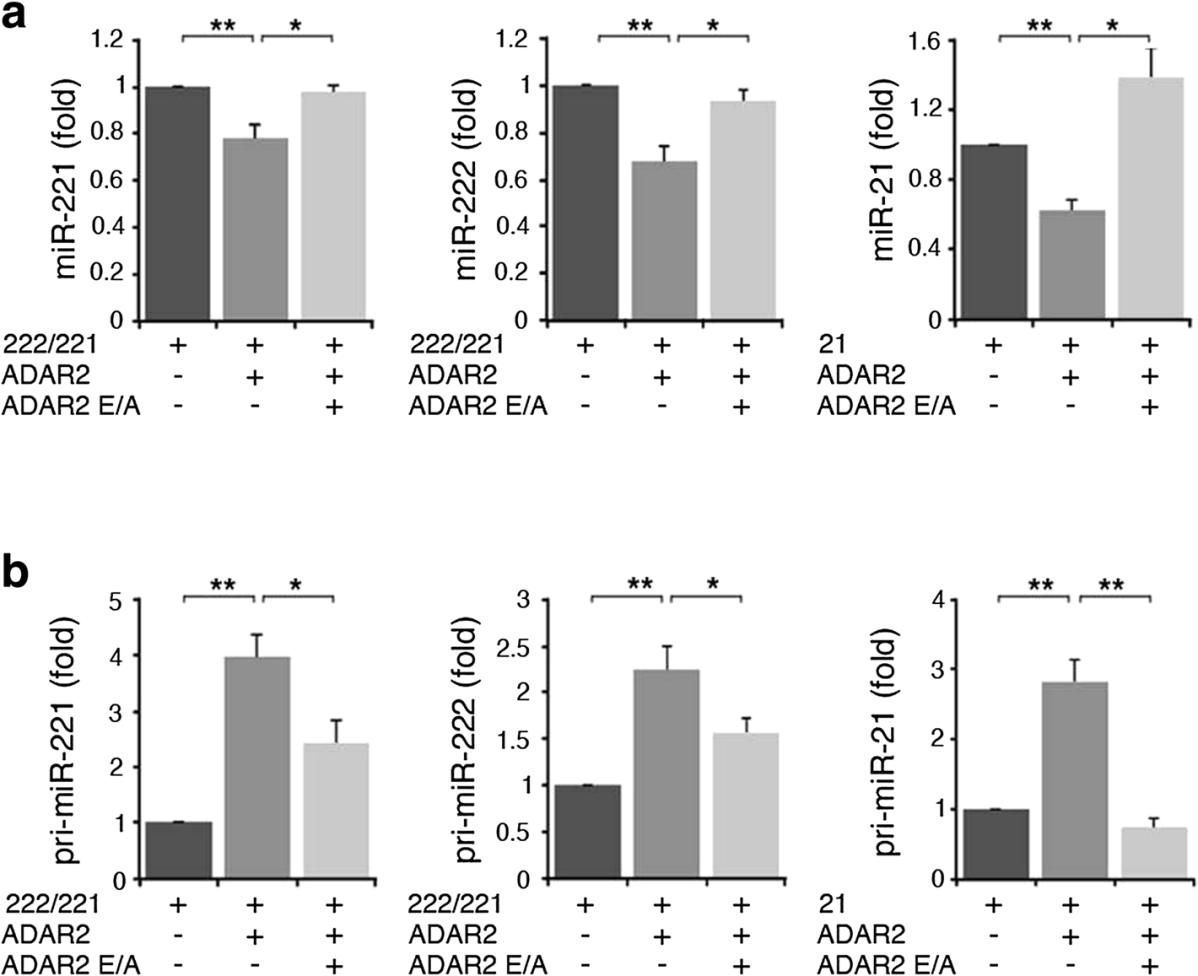
**Modulation of miR-221, miR-222 and miR-21 and their precursors in HEK293T cell lines upon ADAR2 and ADAR2 E/A expression. (a)** Expression levels, using qRT-PCR, of mature miR-221 (left panel), miR-222 (middle panel) and miR-21 (right panel) in HEK293T cells transiently co-transfected with either pri-miR-222/221 or pri-miR-21 plasmids and with ADAR2 or ADAR2 E/A. **(b)** In the same cells, the pri-miR-221 (left panel), pri-miR-222 (middle panel) and pri-miR-21 (right panel) expression levels measured by qRT-PCR are shown. Mature miRNAs and pri-miRNAs were normalized using RNU6B and *GAPDH*, respectively. The expression levels were calculated as a relative-fold increase compared with the sample transfected with the pri-miR-222/221 or pri-miR-21 plasmid arbitrarily set to 1. Mean ± standard error of the mean (n = 3), **P < 0.01, *P < 0.05.

In order to validate our findings in a different cell system, we used HEK293T cells, which show very low ADAR2 editing activity [18]. The cells were transfected with the pri-miR-222/221 cluster or pri-miR-21, either alone or together with the ADAR2 or ADAR2 E/A plasmids (Figure S4 in Additional file 1). Transcripts from these pri-miRNA plasmids had previously been shown to be efficiently processed by mammalian cell machineries in different cell lines [26,29]. We also confirmed these results in HEK293T cells (data not shown). The final amount of mature miRNAs and the corresponding precursors were analyzed 48 h post-transfection by qRT-PCR. As observed in glioblastoma cells and in mouse brain, we found that ADAR2 (but not its inactive version) reduces the levels of mature miR-221, miR-222 and miR-21 (endogenous plus transfected) compared with controls in HEK293T cells (Figure 5a), with the concomitant accumulation of their respective miRNA precursors (Figure 5b). Of note, in this cell system and similarly to mouse brain (Figure 4b), ADAR2 can accumulate pri-miR-21 (Figure 5b), whereas only the pre-miR-21 was affected by ADAR2 activity in glioblastoma cells (Figure 3).

Since the inactive ADAR2 E/A did not alter the expression of these onco-miRNAs (Figures 1, 3 and 5), we hypothesized that possible editing events may occur within pri-miR-221, -222 and -21. To test this possibility, we took advantage of a cell system in which ADAR2 activity was strongly enhanced (HEK293T transiently transfected with ADAR2; Figure S4 in Additional file 1).

RT-PCRs were designed to amplify over-expressed pri-miR-222/221, pri-miR-21 and pri-miR-223 (the latter was used as control) from ADAR2 or ADAR2 E/A HEK293T cells. PCR products were analyzed using three different methods: (i) direct sequencing of cDNA pools, (ii) single-clone sequencing reactions (with 40 to 100 independent clones/sample) and (iii) MiSeq technology (Illumina). Editing events were detected as A-to-G changes in the cDNA sequences. According to the single clone analyses, approximately 20% of each pri-miRNA molecules carry A-to-G changes. Specifically, we found that 28% of the pri-miR-221 clones, 18% of the pri-miR-222 clones and 25% of the pri-miR-21 clones had A-to-G changes.

**Figure 6:**
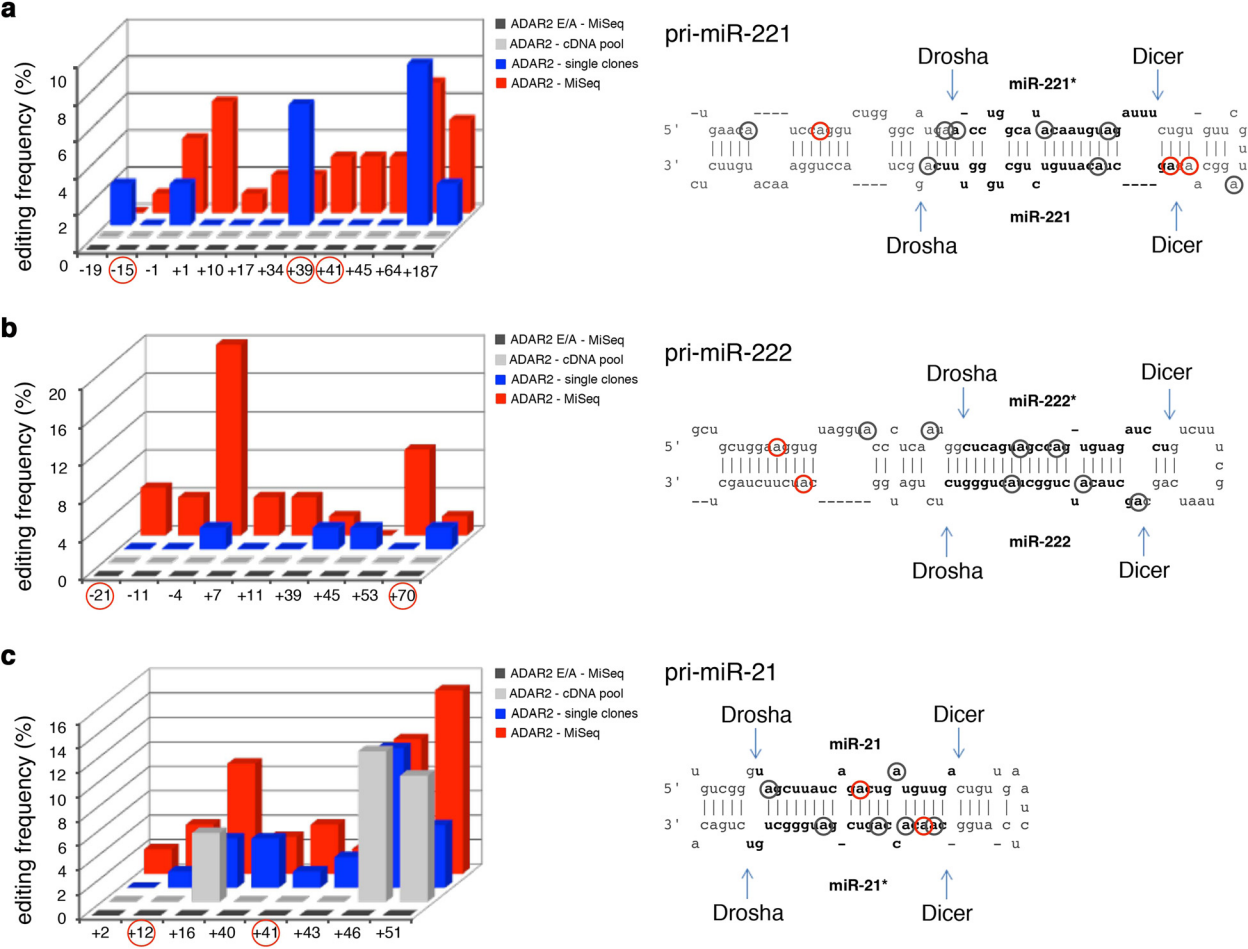
**ADAR2-mediated editing events within pri-miR-221, pri-miR-222 and pri-miR-21 in HEK293T cells. (a-c)** Left: editing sites and percentages of the pri-miR-221 (a), pri-miR-222 (b) and pri-miR-21 (c) are shown. Right: stem-loop structures of each precursor with the edited sites indicated by red and gray circles (the site +187 of pri-miR-221 is not shown in the structure). Adenosines found edited in both ADAR2 HEK293T cells and human brain samples are indicated by red circles. Of note, the site -21 within pri-miR-222 was found highly edited in different human samples, with values increasing from children (4%) to adult (approximately 20%) brain.

Overall, the pri-miR-221 was edited at 12 sites, with the most edited sites being positions -1, +1, +34, +64 and +187 (with editing levels ranging from 4% to 8%; Figure 6a). Pri-miR-222 was found edited at nine sites: the most edited positions were -4 and +53, which were 20% and 9% edited, respectively (Figure 6b). Pri-miR-21 was edited at eight different positions (all located within the pre-miR sequence), with positions +16, +46 and +51 being edited between 9% and 15% (Figure 6c). The A-to-G substitutions we observed are due to ADAR2 activity, and not to RT/sequencing errors/artifacts, as no substitutions were observed in the control ADAR2 E/A cell line (single clone analysis and MiSeq; Figure 6). As a further control, we co-transfected the pri-miR-223 and the ADAR2 plasmids in HEK293T cells in order to test whether ADAR2 can edit any dsRNA structure of miRNA precursors in our over-expressing conditions. No A-to-G changes were found within pri-miR-223 sequences (as tested by single-clone analysis with approximately 70 screened clones, data not shown). Fluctuations in editing percentages detected by different methods are mainly due to their different accuracy/sensitivity, as the sequences analyzed ranged from just a few (cDNA pool), to dozens (single clones), to thousands (MiSeq).

We also investigated editing events in these three pri-miRNAs (pri-miR-222/221 and -21) *in vivo*, using total RNA isolated from human brain tissue (single individual or commercial RNA pools) with similar sequencing technologies. Among the A-to-G changes identified in the over-expressing HEK293T system, some were also found in the human adult and pediatric brain samples. Specifically, A-to-G changes were found within pri-miR-221 (sites -15, +39 and +41), pri-miR-222 (sites -21 and +70) and pri-miR-21 (sites +12 and +41) (Figure 6, adenosines marked with red circles). All these positions showed extremely low percentages of A-to-G changes, ranging from 1.5 to 3% in the different RNA brain samples, with the only exception being site -21 within pri-miR-222, which was highly edited in different human samples: approximately 20% in adult brain (Clontech), approximately 15% in the adult brain pool (Ambion) and approximately 4% in pediatric brain.

None of the A-to-G changes identified in this study have been reported as single nucleotide polymorphisms [30,31]*.*

Edited miRNA precursors can undergo rapid degradation due to the action of specific inosine-dependent ribonucleases, such as Tudor-SN [12,32], and the 2′-deoxythimidine 3′,5′-bisphosphate (pdTp) can inhibit Tudor-SN activity [12]. In order to see if Tudor-SN could degrade our substrates, we examined the pri-miR-221, -222 and -21 sequences (cDNA pools) from ADAR2 HEK293T cells treated with the pdTp inhibitor as previously described [12]. We did not observe any increase in RNA editing levels compared with the control cells in these conditions (data not shown).

**Figure 7:**
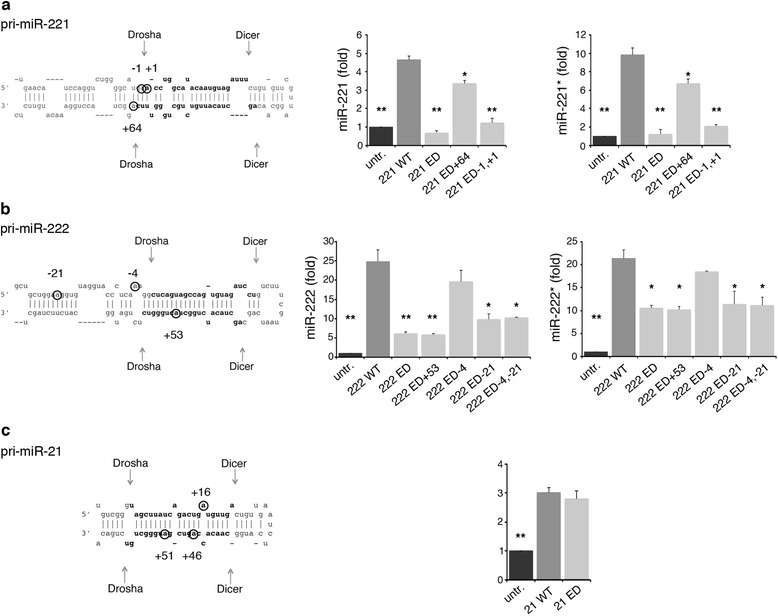
**miRNA maturation of the wild-type and the edited versions of pri-miR-221, pri-miR-222 and pri-miR-21. (a)** Left: the pri-miR-221 sequence structure, indicating the mutagenized/edited positions. Right: the mature miR-221 and -221* levels were measured by qRT-PCR in untreated HeLa cells (untr, black) and in HeLa cells transfected with wild-type pri-miR-221 (221 WT, dark gray), fully edited pri-miR-221 (221 ED, light gray) or pri-miR-221 edited at specific sites (221 ED +64; 221 ED -1,+1). **(b)** Left: the pri-miR-222 sequence structure, indicating the mutagenized/edited positions. Right: the mature miR-222 and -222* levels were measured by qRT-PCR in untreated HeLa cells (untr, black) and in HeLa cells transfected with wild-type pri-miR-222 (222 WT, dark gray), fully edited pri-miR-222 (222 ED, light gray) or pri-miR-222 edited at specific sites (222 ED +53; 222 ED -4; 222 ED -21; 222 ED -4,-21). **(c)** Left: the pri-miR-21 sequence structure, indicating the mutagenized/edited positions. Right: the mature miR-21 levels were measured by qRT-PCR in untreated HeLa cells (untr, black) and in HeLa cells transfected with wild-type pri-miR-21 (21 WT, dark gray) or edited pri-miR-21 (at sites +16, +46, +51) (21 ED, light gray). Mature miRNAs were normalized using RNU6B. The expression levels were calculated as a relative-fold increase compared with the untreated cells and arbitrarily set to 1. Mean ± standard error of the mean (n = 3), **P < 0.01, *P < 0.05 when each sample is compared with the corresponding wild-type pri-miR.

Overall, our data demonstrate that ADAR2 is able to edit the pri-miR-222/221 and the pri-miR-21 transcripts both *in vivo* and in cell lines. In order to verify whether the editing events detected in these three pri-miRs might have an effect on their maturation, we substituted guanosines (by site-specific mutagenesis) within these pri-miR-plasmids at the sites with the highest editing levels as detected by MiSeq analysis (for pri-miR-221: -1, +1, +64; for pri-miR-222: -21, -4, +53; for pri-miR-21: +16, +46, +51) (adenosines marked with circles in Figure 7). The mutagenized and wild-type plasmids were then transfected into HeLa cells. We switched to the HeLa cells as they have an extremely low endogenous level of the mature miR-221, -222 and -21. This thus enabled us to quantify exogenous miRNA expression changes reliably without background noise. We transfected HeLa cells with the wild-type or the edited versions (at single or multiple sites) of each pri-miRNA plasmid and we monitored the expression levels of mature miRNAs as well as miRNAs*. We observed a significant reduction of miR-221 and -222 levels when we used the edited pri-miRNAs versus the unedited ones (Figure 7a, b). Specifically, A-to-G mutagenesis at all the three pri-miR-221 editing sites (-1, +1, +64) strongly inhibited miRNA maturation, with the -1 and +1 sites strongly influencing the maturation process, whereas the +64 site was involved to a lesser extent (Figure 7a). Similarly, the A-to-G mutations of the pri-miR-222 at the -21 and +53 sites play a major role in inhibiting miR-222 maturation, while that at the -4 site does not (Figure 7b). To further confirm that the reduction in mature miR-221 and -222 levels detected after transfection of the edited pri-miRNA plasmids was due to alterations in their processing, we also analyzed the levels of miR-221* and miR-222*, finding similar results (Figure 7a, b).

Surprisingly, no effect was observed when the edited pri-miR-21 plasmid was used (Figure 7c), demonstrating that mutagenesis at these three selected edited sites did not affect miR-21 processing.

### ADAR2 decreases proliferation and migration of glioblastoma cells by inhibiting miR-221, miR-222 and miR-21 maturation

**Figure 8:**
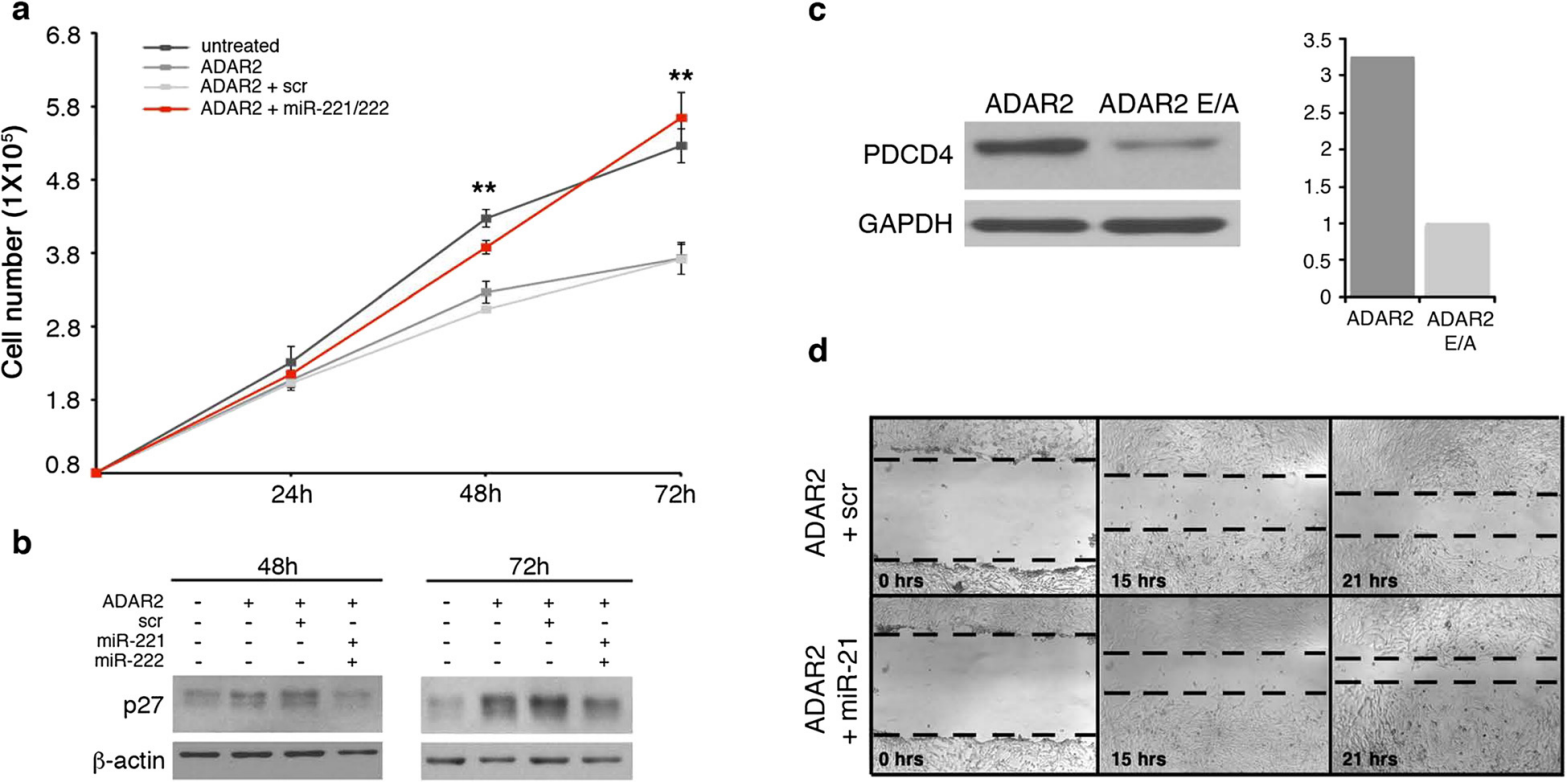
**The ADAR2-mediated anti-tumoral effect is reversed by miR-221, miR-222 and miR-21 expression. (a)** U118 cells (8 × 10^4^; untreated, dark gray), U118 ADAR2 cells (ADAR2, medium gray), and U118 ADAR2 cells transiently transfected with either scramble mimic (ADAR2 + scr, light gray) or with a mix of miR-221- and miR-222-mimic (ADAR2 + miR-221/222, red) were seeded and proliferation was monitored over 3 days. U118 untransfected cells (untreated, dark gray) were used as control. Error bars indicate standard deviations of four independent experiments. Mean ± standard deviation (n = 4), **P < 0.01 when ADAR2 plus miR-221/222 cells (red) are compared with the ADAR2 (dark gray) and ADAR2 plus scramble (light gray). **(b)** Protein lysates were extracted from the cells shown in (a) and analyzed by immunoblotting for p27^Kip1^, a target of miR-221 and miR-222. **(c)** PDCD4 protein analysis after immunoblotting of total protein extracts from U118 ADAR2 and ADAR2 E/A cell lines and the corresponding quantitative densitometric analysis are shown. Each sample was normalized to *GAPDH* and compared with the ADAR2 E/A cells arbitrarily set to 1. A representative sample of two independent experiments is shown. **(d)** Representative photographs of U118 ADAR2 cells transfected with scramble mimic (scr) and miR-21-mimic (miR-21) at 0, 15 and 21 h after scratching the surface of monolayers cells. Only the U118 ADAR2 plus miR-21 cells show an increase in motility when compared with the control cells (scr). The wound healing assay was performed in a time interval in which the cells do not divide (data not shown).

We have previously shown that editing activity mediated by ADAR2 in glioblastoma cells decreases cell proliferation and migration both *in vitro* and *in vivo*, while glioblastoma cells transfected with the inactive ADAR2 E/A enzyme retain malignant features similar to the untreated cells [18,21]. In order to test whether the decrease in miR-221, -222 and -21 levels mediated by ADAR2 reduces cell aggressivity, we over-expressed miR-221, -222 or -21 in ADAR2 glioblastoma cells and evaluated their effects on cell proliferation and migration, generally affected by these three miRNAs. We transiently transfected an equal amount (100 nM) of scramble or miR-221- plus miR-222-mimic in U118 ADAR2 cells (Figures S5a in Additional file 1). We monitored cell proliferation of the untransfected and transfected U118 cells over four days, observing that while the presence of ADAR2 alone inhibited cell proliferation compared with the untreated cells, the combination of the two miRNAs considerably increased cell proliferation (at 48 to 72 h post-transfection; Figure 8a). Next, we assessed the protein expression levels of a well-known target of miR-221 and -222, p27^Kip1^ [33]. This protein is down-expressed in glioblastomas and plays a pivotal role at the G1/S cell cycle checkpoint [34]. As expected, the level of p27^Kip1^ decreased upon miRNA-mimic transfection compared with U118 ADAR2 scramble or U118 ADAR2 cells (Figure 8b). Similar results were also observed in another glioblastoma cell line (A172) (Figures S5c and S6a,b in Additional file 1).

Of note, the U118 ADAR2 E/A cells (which have a high level of both miR-221 and miR-222 and a high proliferative rate) did not show cell proliferation increases comparable to that observed in the ADAR2 cells when they were transfected with miR-221- plus miR-222-mimic (Figures S7 in Additional file 1). This effect is probably due to the high levels of these miRNAs in highly malignant glioblastoma cells [35] that are thus not ‘corrected/decreased’ by the ADAR2 activity. These data indicate that the reduction of miR-221 and -222 levels mediated by ADAR2 editing activity contributes significantly to the inhibition of glioblastoma proliferation.

miR-21 is involved in cell proliferation and cell migration [36]. Among the validated miR-21 target genes, *PDCD4* plays an important role in cancer cell migration [37]. In our previous study, we showed that ADAR2 inhibits glioblastoma cell migration [18]. Here, we demonstrate that the expression of ADAR2 in glioblastoma cells can significantly down-modulate miR-21 and up-regulate PDCD4 protein levels in the U118 (Figure 8c) and A172 (Figures S6c in Additional file 1) cell lines. To evaluate whether miR-21 transfection can abolish the inhibition of cell migration caused by ADAR2, we over-expressed either miR-21-mimic or scramble (100 nM) in U118 and A172 ADAR2 cells (Figures S5b, d in Additional file 1). We monitored cell migration by wound healing assay over 24 h, a time interval in which the cells do not divide (data not shown). We observed that cell migration increases in both U118 (Figure 8d) and A172 (Figures S6 in Additional file 1d) cell lines upon miR-21 over-expression. Altogether, these data indicate that ADAR2 exerts its anti-proliferative and anti-migratory activity in glioblastoma also through the modulation of miR-221, miR-222 and miR-21.

## Discussion

It is becoming clear that a large number of RNA-binding proteins join forces to create an additional layer of complexity in miRNA maturation and function. It is thus critical that we gain a better understanding of the mechanisms by which they control miRNAs and affect normal physiology and disease [38].

Herein, we show that ADAR2, an essential RNA-binding protein that converts A-to-I in dsRNAs, plays a critical role in controlling miRNA expression levels and determining their final sequence. By analyzing normal brain and glioblastoma tissues as well as ADAR2-modified glioblastoma cells, we found that: 1) selected miRNAs undergo ADAR2-mediated editing in normal brain, some of which is within the seed sequence; 2) editing within miRNAs is decreased (or lost) in glioblastoma, where ADAR2 activity is impaired, compared with normal brain; 3) glioblastomas have altered miRNA expression profiles when compared with normal brain; 4) the rescue of ADAR2 activity in glioblastoma cells restores the edited miRNA population and tends to rebalance the miRNA expression profile (onco-miRNAs versus tumor suppressor miRNAs) towards a state resembling normal brain tissue; 5) the most striking effect of ADAR2 rescue in glioblastoma cells is a general decrease in the levels of several onco-miRNAs (such as miR-221, -222, -21); 6) ADAR2 can edit miR-222/221 and miR-21 precursors and decrease the expression of the corresponding mature miRNAs both *in vivo* (mouse brain) and in human glioblastoma cells, and this has significant effects on cell proliferation and migration.

In our previous study, we demonstrated that ADAR2 activity is progressively lost during the progression of astrocytoma malignancy grade in children (from grade I to grade IV) and that this contributes to cancer progression [18]. Here, we show that ADAR2 activity is fundamental for miRNA homeostasis and we identify ADAR2 as an important factor for miRNA expression and function in both normal and cancer cells.

The most important information that comes from ADAR2 rescue in glioblastoma cells is that this enzyme can modulate the expression of a large number of miRNAs. The general tendency observed is that ADAR2 limits the expression of many miRNAs and most especially of onco-miRs. This finding is intriguing, as ADAR2 may thus have the opposite effect to the other RNA editing enzyme, ADAR1, which has recently been shown to enhance miRNA processing [14].

Among the onco-miRNAs down-expressed by ADAR2, we focused on miR-221, miR-222 and miR-21. These miRNAs are often over-expressed in tumors, including glioblastoma [6]. We show that ADAR2 activity inhibits miR-221, -222 and -21 maturation and causes accumulation of their precursors in different cell lines (including glioblastoma cells) and in normal mouse brain. Consist with this, we found a significant increase of miR-221, -222 and -21 levels in *Adar2*^*-/-*^ mouse brain, with a concomitant decrease of the corresponding precursors. Editing of miRNA precursors may impair the production of mature miRNAs [11]. Considering that the inactive ADAR2 E/A did not alter miRNA expression (miR-221, -222 and -21), we tested whether editing events may occur within their pri-miRNAs. Using an over-expressing cell system (HEK293T), we identified multiple editing sites (with editing levels ranging from 2% to 20%) within the stem-loop structure of pri-miR-222/221 and pri-miR-21, but not within the control pri-miR-223. The integrity of the dsRNA stem structure of miRNA precursors is essential for their maturation [2]. Most of the identified A-to-I editing events replace an A-U or U-A Watson-Crick pair with a less stable I**∙**U or U**∙**I wobble pair, leading to changes in the stem structure and/or stability. Investigating the miRNA maturation process of wild-type and the edited versions of pri-miR-222/221, we found that editing at the chosen positions did indeed affect miRNA processing, since both miRNA and miRNA* expression levels were impaired. Conversely, the mutagenized/edited pri-miR-21 (carrying three A-to-G changes) was processed normally, indicating that the selected sites were not involved in miR-21 maturation, at least in our system. Previous studies reported that not all the editing events in pri-miRNA affect Drosha cleavage [12]. Indeed, we show that only specific editing events (or combinations of events) within miRNA precursors affect miRNA maturation.

We have to underline that ADAR2 can edit multiple substrates *in vivo*; therefore, we cannot exclude the possibility that ADAR2 may control miRNA expression not only by editing miRNA precursors directly but also by editing other RNAs involved in miRNA maturation.

Some of the editing sites within pri-miR-222/221 and pri-miR-21 identified in the ADAR2 HEK293T cells were also detected in normal human brain, even if at low percentages compared with the cell system. Only site -21 within pri-miR-222 was found to be highly edited in different human brain samples, with editing increasing from child (4%) to adult (approximately 20%) brain. Interestingly, this editing position alone (which is located in the lower stem of pri-miR-222 in the extension of the pre-miRNA structure [39] and distant from the Drosha cleavage site) is able to hamper miR-222 maturation by more than 50% (Figure 7b). Also of note, miR-222 decreases its expression during porcine brain development from embryonic to adult cortex [40]. Overall, our findings indicate a connection between ADAR2-mediated editing of miR-222 and brain development.

The generally low editing frequencies observed within the three precursors in this study *in vivo* could be due to the action of inosine-dependent nucleases, such as Tudor-SN, that can degrade edited miRNA precursors [12,32]. However, the inhibition of Tudor-SN by pdTp did not increase editing levels in our cell system (HEK293T), in contrast to previous reports on other miRNA precursors in HEK293T cells [12]. However, a recent study demonstrated the existence of a novel inosine-dependent nuclease [41]; therefore, we cannot exclude the possibility that different inosine-dependent nucleases may play a role in the fate of specific edited miRNA precursors *in vivo*, such as the ones identified in this study.

**Figure 9:**
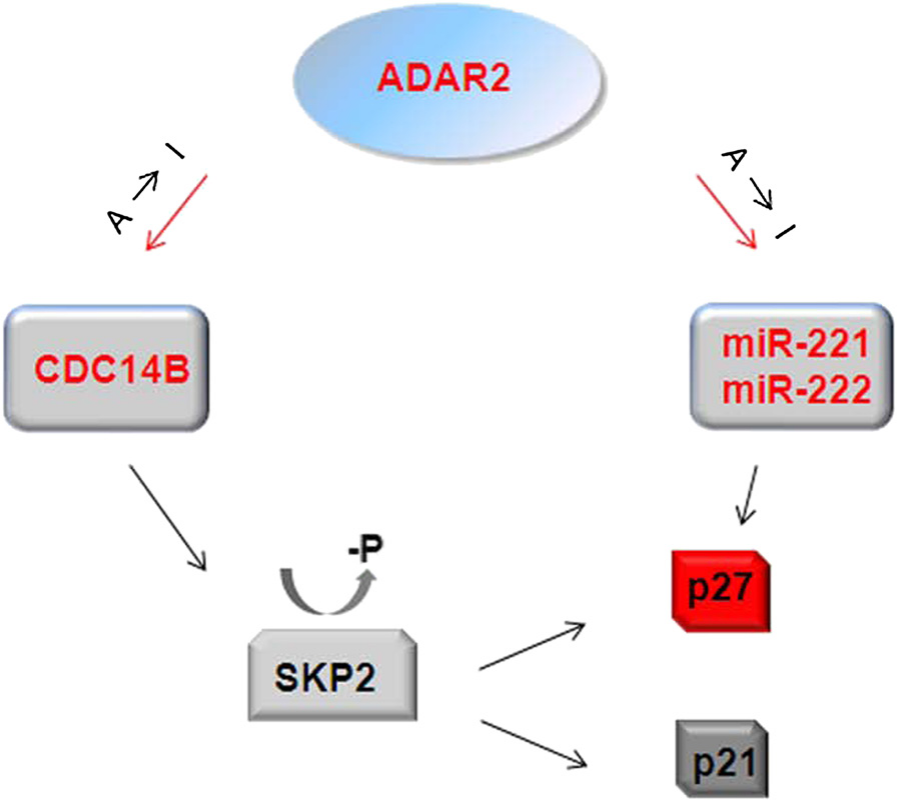
**Cartoon summarizing the role played by ADAR2 over p27** ^**Kip1**^ **.**

We previously showed that ADAR2 rescue in glioblastoma cells has important anti-tumoral effects, such as the inhibition of cell proliferation and migration [18,21]. It is conceivable that ADAR2 also exerts its anti-tumoral effects through miRNA modulation, such as of miR-221, miR-222 and miR-21. In order to explore this possibility, we reintroduced miR-221 and miR-222 into ADAR2-modified glioblastoma cells (U118 and A172) and analyzed cell proliferation. The over-expression of miR-221 plus miR-222 caused a significant boost in cell proliferation and abolished the anti-tumoral effect of ADAR2. These data indicate that miR-221 and miR-222 are important mediators of the effects of ADAR2, through which this enzyme plays its anti-proliferative role in glioblastoma cells. In our previous study, we identified the *CDC14B* transcript as an essential ADAR2 target gene, acting over the Skp2/p21/p27 pathway, whose editing slows down cell proliferation [21]. Here, we found that two miRNAs (miR-221 and miR-222) that act in the same molecular pathway (through p27^Kip1^) are also modulated by ADAR2 activity, indicating that this pathway is particularly sensitive to epigenetic mechanisms such as RNA editing (Figure 9).

We also previously showed that ADAR2 plays an important role in inhibiting glioblastoma cell migration [18]. Here, we demonstrate that ADAR2 strongly down-modulates miR-21 and up-regulates PDCD4 protein (*PDCD4* is a validated miR-21 target gene [37]) and that this has important consequences in inhibiting glioblastoma cell migration (Figure 8c,d and S6c,d in Additional file 1).

Our data add an important piece of information regarding miRNome fluctuation due to ADAR2 in glioblastomas, indicating ADAR2 as an important player in gliomas. Indeed, ADAR2-mediated RNA editing is strongly dysregulated in glioblastoma cell lines (derived from adult *de novo* glioblastoma [42]), in pediatric [18,21,43] as well as adult gliomas [19,20]. However, adult and pediatric gliomas are very different tumors at both the genomic and epigenomic level. For example, differences in cell methylomes were observed between pediatric and adult gliomas [44]. In this respect, mutations in the isocitrate dehydrogenase (IDH) 1 and 2 genes, which are able to alter the methylome [45], were observed in adult and secondary glioblastomas [46], but not in pediatric or *de novo* glioblastomas [45]. In order to see if specific ADAR2 genetic alterations occurred in gliomas, we analyzed the *ADAR2* gene (21q22.3), interrogating different available datasets [47-49]. We did not identify any somatic aberrations in ADAR2; therefore, it is more likely that epigenetic/post-transcription events are responsible for its inactivation during gliomagenesis.

Future studies, including a comprehensive analysis of molecular pathways and of ADAR2-mediated RNA editing profiles, should further provide important information about changes in genetic, epigenetic and post-transcriptional mechanisms among different groups of gliomas.

## Conclusions

**Figure 10:**
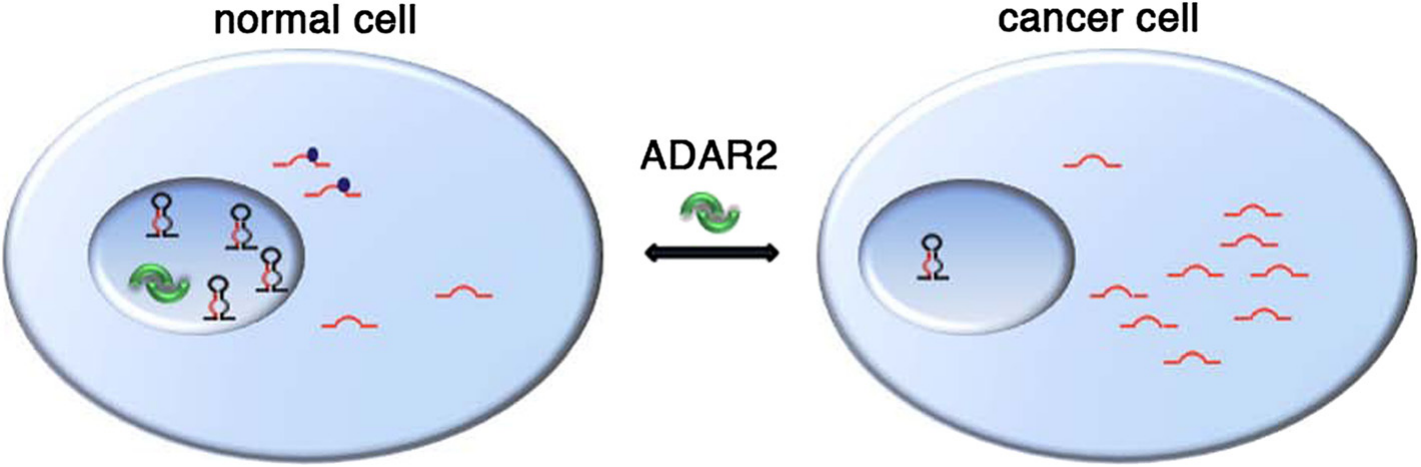
**Cartoon summarizing the role played by ADAR2 in cancer versus normal cells/tissues.** ADAR2 editing activity rebalances miRNA expression and recovers the edited miRNA population. Dark blue dots represent editing events present in normal brain but absent in glioblastoma.

We propose ADAR2 as a ‘radar’ enzyme that maintains a degree of editing in the miRNA population and balances miRNA expression, maintaining them at physiological, that is, safe, levels. Whenever ADAR2 is impaired (that is, in glioblastoma), miRNA homeostasis is altered and this may contribute to cancer progression (Figure 10). In summary, our findings identify ADAR2 as a promising target for an innovative anti-tumoral strategy, since ADAR2 alone can simultaneously modulate more than one miRNA and cellular pathway altered in cancer cells. Future studies on dissecting the causes of ADAR2 deregulation will be aimed at the identification of compounds that can adjust ADAR2 expression/activity in glioblastoma and provide a potential anti-cancer therapy.

## Materials and methods

### Cell lines

Well-characterized permanent human glioblastoma cell lines U118 MG (HTB-15™) and A172 (CRL-1620™), derived from adult patients with malignant *de novo* glioblastoma [42], were obtained from American Type Culture Collection (ATCC). Human embryonic kidney 293 T (HEK293T, CRL-1573™) and human cervical cancer (HeLa, CCL2™) cells were obtained from ATCC. All the cell lines were routinely maintained in Dulbecco’s modified Eagle’s medium (DMEM) supplemented with 10% fetal bovine serum (Gibco-Life Technologies, Carlsbad, CA, USA), 100 U/ml penicillin and 100 μg/ml streptomycin, at 37°C in 5% CO_2_. U118 and A172 cell lines stably over-expressing the active EGFP-ADAR2 or the inactive EGFP-ADAR2 E/A enzyme were generated as previously reported [18,21]. The U118 ADAR2 cell line stably silenced for ADAR2 (siADAR2) and the scramble were generated as previously described [21]. All the stable cell lines used in this study were polyclonal to avoid problems due to positional insertion effects.

### Human and mouse brain tissues/RNAs

Brain tissues from homozygous *Adar2* knockout and control mice were kindly supplied by Dr Michael Jantsch. Mouse tissues were obtained in accordance with the local (Austria) research ethics regulations. We used total RNA of human adult brains from a pool of 23 individuals (AM6050, Ambion-Life Technologies, Carlsbad, CA, USA) and from an 18-year-old single donor (636530, Clontech, Mountain View, CA, USA). Furthermore, we used normal human brain tissue obtained from a pediatric donor (undergoing focal brain resection for head injury sequelae) and a *de novo* glioblastoma (grade IV astrocytoma) tissue. The study was revised and approved by the local institutional review board (study number 571/2012) of Bambino Gesù Children’s Hospital of Rome. Informed consent from patients was obtained for the use of biological samples for research purposes.

### RNA isolation

The small (up to 200 nucleotide) and total RNA fractions were isolated using miRVana™ miRNA Isolation Kit (Ambion-Life Technologies) and TRIzol reagent (Invitrogen-Life Technologies, Carlsbad, CA, USA), respectively. Both procedures were performed according to the manufacturer’s recommendations. RNA concentration and purity (A260/A280nm ratio) were evaluated using NanoDrop ND-2000 (Thermo Scientific, Walthman, MA, USA). RNA quality was assessed by gel electrophoresis or by an Agilent 2100 Bioanalyzer microfluidics-based platform (Agilent Technologies, Santa Clara, CA, USA) with two chips: Agilent RNA 6000 Nano Kit for total RNA and Agilent Small RNA kit for low molecular weight RNA. RNA samples were DNase treated (Ambion-Life Technologies).

### miRNA microarray

Microarray experiments were performed using a miRCURY™ LNA microRNA Array Power Labeling Kit (Exiqon, Vedbaek, Denmark). Total RNA from U118, U118 ADAR2 and U118 ADAR2 E/A were labeled with specific fluorescent dyes (Hy3 and Hy5), following Exiqon’s protocol. Fluorochrome-labeled RNA samples were then combined, denatured and hybridized on homemade slides, containing LNA-modified microRNA capture probes targeting all human miRNAs listed in miRBASE v.8.1 [50]. The hybridization was performed according to the miRCURY™ LNA array manual using hybridization chambers (Agilent Technologies), for 16 h at 56°C. A ScanArray Lite Microarray Scanner (Packard Bioscience Company, Arvada, CO, USA was used to acquire images and the software GenePix Pro 6.0 was used to quantify hybridization signals. Microarray images were processed and analyzed using Genepix Pro 6.0, Excel and TIGR Multiexperiment Viewer v.4.0. Data were normalized using different endogenous controls present in the LNA-modified spotted library. The Hy3/Hy5 ratios of ADAR2 versus ADAR2 E/A were log2-transformed and data from three independent experiments were averaged. To identify the miRNAs with a statistically significant difference of expression, we used the Benjamini-Hochberg multiple-testing correction for the *t*-test results. We adopted a FDR cutoff of 0.2 (FDR ≤ 0.2) that ensures a global statistical accuracy of 80%. We selected only miRNAs with a log2(ratio) above +0.5 or below -0.5.

### miRNA deep-sequencing: editing and expression profiling

miRNA capture and library construction were conducted using Illumina’s TruSeq Small RNA Sample Prep Kit according to the manufacturer’s protocol (Illumina, San Diego, CA, USA). The mature miRNA libraries were sequenced with barcodes on one lane of an Illumina HiSeq2000 instrument following the manufacturer’s protocol. The total number of reads was 16.8 million, 35.6 million, 21.6 million and 36.5 million for the U118 untreated, ADAR2, ADAR2 E/A and siADAR2 cell lines, respectively, and 10 million, 40 million and 124 million for the Ambion pool of adult brains, frontal lobe sample and glioblastoma sample, respectively. All reads were filtered such that the quality of each read will not be below a given threshold value (chosen to be 20) in more than three positions. In addition, sequences identified as 5′ or 3′ adaptors were removed. After adaptor trimming, reads longer (>28 bases) or shorter (<15 bases) than the typical length of a mature miRNA were also removed. Editing events were identified as previously described [23]. Briefly, considering that the 3′ end of mature reads undergoes extensive modifications [51], the last two bases of the read were trimmed [52]. The filtered and trimmed reads were aligned using Bowtie [53] against the human genome (UCSC hg19/GRCh37) allowing up to one mismatch with quality score (phred score) of 30 and above. The total number of reads aligned to known miRNAs (miRBase, release 17) [54] was 4.3 million, 6.7 million, 6.6 million and 6.3 million for U118 untreated, ADAR2, ADAR2 E/A and siADAR2 cells, respectively, and 1.4 million, 3.2 million and 1.1 million for Ambion’s pool of adult brains, frontal lobe sample and glioblastoma sample, respectively. All the locations in each mature or miRNA* were screened for mismatches that were overrepresented considering the expected sequencing error rate of 0.1% (as only mismatches with phred score of 30 were allowed). This was done by applying the binomial cumulative distribution on the counts of each sequenced nucleotide. All the miRNA expression profiles were normalized and statistically significant differences between the profiles were identified as described previously [25]. Briefly, the expression profiles were normalized using a variation of the trimmed mean of M-values normalization method [25,55]. Then, we looked for expression differences that cannot be explained by the expected Poisson noise with *P*-value <0.05 and Bonferroni correction for multiple testing. Fold-changes between counts of sample A and B were calculated using the formula log2[(A + 1)/(B + 1)], in order to avoid problems associated with zero values.

### Quantitative real-time-PCR

qRT-PCRs were performed to validate the expression of specific mature miRNAs, using pre-designed stem-loop primers (TaqMan MicroRNA Assay, Applied Biosystems-Life Technologies). cDNA was synthesized from 10 ng of total RNA using TaqMan MiR Reverse Transcriptase Kit (Applied Biosystems-Life Technologies) according to the manufacturer’s instructions.

For pri-miRNA and mRNA, 1 μg of total RNA (pre-treated with DNase I) was used to generate cDNA by the ImProm-II Reverse Transcription System (Promega, Madison, WI, USA) using random hexamer primers according to the manufacturer’s instructions.

Custom stem-loop primers were designed for the detection of pre-miRNAs (Applied Biosystems-Life Technologies). A pre-amplification step was introduced for the detection of pre-miRNAs to increase the sensitivity of the following real-time PCR analysis [56]. Briefly, the pre-amplification reaction was carried out using Taqman PreAmp Master Mix (Applied Biosystems-Life Technologies) and the following cycling conditions: 95°C for 10 minutes, 14 cycles of 95°C for 15 s and 60°C for 4 minutes, followed by a hold at 4°C. qRT-PCRs were conducted on an ABI PRISM 7700 Sequence Detection System (Applied Biosystems-Life Technologies) using TaqMan Universal PCR MasterMix. The small endogenous nuclear RNA U6 (RNU6B) and *GAPDH* were used as controls for normalization of mature miRNAs/pre-miRNAs and mRNAs/pri-miRNAs, respectively. The relative amount of each substrate was calculated by the 2^-ΔΔCT^ method [57]. Expression levels were represented as a relative-fold increase compared with the control sample arbitrarily set to 1. All qRT-PCRs were performed in duplicates and repeated at least two times from independent RT-PCRs. All the primers were supplied by Applied Biosystems: miR-221, ID 000524; miR-221*, ID 002096; miR-222, ID 002276; miR-222*, ID 000525; miR-21, ID 000397; miR-223, ID 002295; RNU6B, ID 001093; pri-miR-221, ID Hs03303007_pri and Mm03307181_pri; pri-miR-222, ID Hs03303011_pri and Mm03307187_pri; pri-miR-21, ID Hs03302625_pri and Mm03306822_pri; *ADAR2*, ID Hs00953730_m1; *GAPDH*, ID Hs99999905_m1; *Actb*, ID Mm00607939_s1.

### RNA editing analysis by Sanger sequencing

For the editing analysis, RNA samples were pretreated with DNase I and cDNAs were generated with Superscript II Reverse Transcriptase (Invitrogen-Life Technologies) using random hexamer primers or transcript-specific oligonucleotides, according to the manufacturer’s instructions. The cDNAs were amplified by PCR reactions using Expand high fidelity Plus PCR System (Roche, Basel, Switzerland) and specific primers. The specific PCR products were gel purified (Qiaquick, Qiagen, Venlo, Limburgo, Netherlands), directly sequenced or cloned into pGEM T-easy vector (Promega) and transformed into *Escherichia coli*.

Direct sequencing (ABI 3500 Genetic Analyzer, Applied Biosystems-Life Technologies) was performed on cDNA pools and editing levels were calculated as previously described [18,58]. Briefly, editing was quantified dividing the area under the curve (AUC) of the G peak by the sum of the AUC of A and G peaks of the analyzed site. For single clone analysis, approximately 40 to 100 individual cDNA clones were sequenced for each sample using T7 or Sp6 primers and A-to-G changes in the individual clones were analyzed. All primer sequences used for these studies are available on request.

### RNA editing analysis by MiSeq technology

The specific PCR products of pri-miR-221, pri-miR-222 and pri-miR-21 from different samples were gel purified, quantified and used in equimolar amounts. Dual-indexed paired-end libraries for subsequent cluster generation and DNA sequencing of amplicon pools were prepared using Illumina Nextera®XT DNA Sample Preparation Kit (Illumina), as recommended in the manufacturer’s instructions. MiSeq sequencing of the sample libraries was performed using the MiSeq Reagent Nano Kit v2 (300 cycles) and analysis of the reads produced was performed by IGV (Integrative Genomics Viewer) software [59].

### Northern blot

For northern blot analysis of miRNAs, 20 μg of total RNA was separated on 10% denaturing polyacrylamide gels, electroblotted onto Immobilon Nylon^+^ membrane (Millipore Corp., Billerica, MA, USA) and UV-crosslinked. The specific probes were end-labeled using T4 polynucleotide kinase and [γ-^32^P] ATP. Oligonucleotide probes, corresponding to the antisense miRNA sequences used, were: miR-221 probe, 5′-gaaacccagcagacaatgtagc-3′; miR-222 probe, 5′-gagacccagtagccagat-3′; miR-21 probe, 5′-tcaacatgagtctgataagcta-3′; and U6 probe, 5′-cacgaatttgcgtgtcatccttgcgcaggggcc-3′. Hybridization was done at 37°C in 0.1% SDS, 5X Denhardt’s and 6X SSPE overnight and membranes were washed at 42°C with 6X SSPE. Membranes were stripped by boiling in 0.1% SDS and rehybridized. U6 RNA was used as control.

### Immunoblotting

Total protein extracts were isolated with RIPA lysis buffer in the presence of a protease inhibitor mixture and phosphatase inhibitor cocktail (Sigma-Aldrich, St. Luis, MO, USA). Protein extracts were quantified with a BCA Protein Assay Kit (Pierce Biotechnology, Rockford, IL, USA). Equal amounts of total cellular lysates (30 μg) were separated by SDS-PAGE, transferred on nitrocellulose membrane, analyzed by immunoblotting with the appropriate antibodies and then revealed by ECL (enhanced chemiluminescence) (GE Healthcare, Buckinghamshire, UK). The antibodies used in this study were: anti-p27 (1:500; Cell Signaling, Danvers, MA, USA), anti-PDCD4 (1:1,000; Origene Technologies, Rockville, MD, USA), anti-ADAR2 (1:200; Sigma), anti-β-actin (1:5,000; Santa Cruz Biotechnology, Santa Cruz, CA, USA) and anti-GAPDH (1:5,000; Cell Signaling). The protein-specific signals were quantified by densitometric analysis using ImageJ v1.47 software.

### Plasmid constructs and cell transfection

The pri-miR-222/221 cluster sequence was amplified by PCR from genomic human DNA using the following primers: miR-222/221 sense, 5′-cgcagatcttttcttccacagagcccctcc-3′; miR-222/221 antisense, 5′-gctcgaggcggtcctttctctgcactct-3′. The correct sequences of amplified products were verified by sequencing and cloned into the BamHI-XhoI sites of pCDNA(+)3.1 vector. The pCMV-miR-21 vector was obtained from Origene Technologies. The plasmid containing the pri-miR-223 sequence was kindly provided by Dr Alessandro Fatica (Sapienza University, Rome). EGFP-ADAR2 and EGFP-ADAR2 E/A constructs were generated as previously described [18].

HEK293T cells seeded into a six-well plate were transiently co-transfected at 80% confluence using Lipofectamine 2000 (Invitrogen-Life Technologies) with either 2 μg of EGFP-ADAR2 or EGFP-ADAR2 E/A in the presence of 1.5 μg of pri-miR-222/221 or pri-miR-21 plasmid. After 48 h, the cells were collected and analyzed.

### Site-directed mutagenesis to generate edited pri-miR-221, -222 and -21

A-to-G single point mutations in the pri-miR-221, -222, -21 sequences were introduced using a site-directed mutagenesis kit (Agilent Technologies) following the manufacturer’s instructions. The oligonucleotides used for the mutagenesis are available on request.

### miRNA mimic transfection

One day before transfection, U118 and A172 cells (8 × 10^5^/well) were plated into a six-well plate. miRIDIAN miRNA mimics (small, chemically modified dsRNAs that mimic endogenous miRNAs; Dharmacon-GE Healthcare, Lafayette, CO, USA) miR-221, miR-222 or miR-21 (100 nM or 200 mM) were transfected into cells using Oligofectamine (Invitrogen-Life Technologies), according to the manufacturer’s instructions, and then tested for *in vitro* proliferation and motility.

### Proliferation assay

The day after transfection, cell viability (trypan blue dye exclusion) was determined daily, from day 1 to day 3.

### Monolayer wounding assay

For evaluation of *in vitro* motility, a monolayer wounding (scratch) assay was performed. Cells were allowed to form a monolayer on a culture dish surface and, when approaching 100% cell confluence, a wound was made by scratching the monolayer with a pipette tip. After the scratching, the cells were incubated in a 5% CO_2_ incubator at 37°C for further 24 h. Photographs of the wound were taken at various time points after wounding. Two independent series of experiments were performed.

### Data availability

The miR-seq data were deposited to the Sequence Read Archive (SRA), under the following accession codes: SRX735409 (U118 ADAR2), SRX735410 (U118 ADAR2 E/A), SRX764455 (U118 siADAR2), SRX039177 (brain Ambion), SRX747635 (glioblastoma). The miR-array data discussed in this publication have been deposited in NCBI’s Gene Expression Omnibus (GEO) and are accessible through GEO Series accession number GSE63694.

The manuscript’s experimental methods comply with the Helsinki declaration.

## Additional files

Additional file 1: Figures S1 to S7. Supplementary Figures S1 to S7 and their corresponding figure legends in portable document format. Additional file 2: Table S1. Mature miRNAs with significant change in expression levels [log2(ratio)] as a result of ADAR2 and ADAR2 E/A over-expression in U118 cell line (by miRNA array). Additional file 3: Table S2. Mature miRNAs with statistically significant changes in expression levels (log2(ratio) of at least ±0.5) between U118 ADAR2 versus U118 ADAR2 E/A and which inverted their expression trend in the U118 siADAR2 cell line. Additional file 4: Table S3. Mature miRNAs with significant changes in expression levels (log2(ratio) of at least ±0.5) between normal human brain versus glioblastoma.

## Abbreviations

ATCC: American Type Culture Collection
dsRNA: double-stranded RNA
FDR: false discovery rate
miRNA: microRNA
pdTp: 2′-deoxythimidine 3′,5′-bisphosphate
pre-miRNA: precursor miRNA
pri-miRNA: primary miRNA
qRT-PCR: quantitative real-time PCR
