# Supplementary material for: Modulation of microRNA editing, expression and processing by ADAR2 deaminase in glioblastoma
Source: Genome Biol. 2015 Jan 13;16(1):5. doi: 10.1186/s13059-014-0575-z (PMC4326501; doi:10.1186/s13059-014-0575-z)
Supplement: Additional file 1: Figures S1 to S7. — Supplementary Figures S1 to S7 and their corresponding figure legends in portable document format. [file 13059_2014_575_MOESM1_ESM.pdf]

Supplementary Figure Legends

Figure S1. ADAR2 and ADAR2 E/A protein expression in U118 glioblastoma cell lines. ADAR2 immunoblotting of total protein extracts from U118 ADAR2 and U118 ADAR2 E/A stably transfected cell lines (left panel) and the corresponding quantitative densitometric analysis (right panel) are shown. Each sample was normalized to GAPDH and compared to the ADAR2 cells arbitrarily set to 1. One representative of 3 independent experiments is shown. Mean ± s.d. (n=3).

Figure S2. Northern blotting of total RNA isolated from glioblastoma cell lines (U118 and A172) stably transfected with ADAR2 or ADAR2 E/A enzyme. (**a**) RNA from U118 cell lines was blotted and antisense probes of miR-221, miR-222 and miR-21 were used. Small nuclear RNA U6 was included as control. One representative of 3 independent experiments is shown. (**b**) The same experiment showed in (**a**) was performed in the A172 cell lines.

Figure S3. Northern blotting of total RNA from U118 ADAR2 and ADAR2 E/A in order to detect the pre-miR-21. Small nuclear RNA U6 was used as control.

Figure S4. Controls of the transient cotransfection experiments in HEK293T cells with either pri-miR-222/221 cluster or pri-miR-21 plasmid and ADAR2 or ADAR2 E/A. (**a**) qRT-PCR analysis of *ADAR2* mRNA levels in HEK293T cells transfected with the pri-miR-222/221 cluster (left panel). The right panel shows a semi-quantitative analysis of the pri-miR-222/221 cluster plasmid (vector-specific primers), with β-actin used as control. (**b**) The same experiments showed in (**a**) were performed in the HEK293T cells transiently transfected with pri-miR-21 plasmid. The expression levels of *ADAR2* were calculated as a relative-fold increase compared to the HEK293T plus pri-miR-222/221 or pri-miR-21 alone and arbitrarily set to 1. Each sample was normalized to *GAPDH* mRNA. Mean ± s.e.m. (n=2).

Figure S5. Transfection efficiency of miRNA mimics in ADAR2-U118 and ADAR2-A172 glioblastoma cell lines. (**a,b**) qRT-PCRs of miRNA-mimics performed in U118 ADAR2 cells transiently transfected with scramble (scr, 100nM) and miR-221-mimic plus miR-222-mimic (100nM) or with miR-21-mimic (100nM). (**c,d**) The same experimental controls were performed in the A172 ADAR2 cell line. Expression levels were calculated as a relative-fold increase compared to the ADAR2 untransfected cells arbitrarily set to 1. Each sample was normalized to RNU6B levels. Mean ± s.e.m. (n=2).

Figure S6. Reversion of ADAR2-mediated anti-tumoral effects by miR-221, miR-222 and miR-21 transfection in the A172 ADAR2 cell line. (**a**) $8x10^4$ A172 cells stably overexpressing ADAR2 (ADAR2, middle grey) and transiently transfected with either scramble mimic (ADAR2+scr, light grey) or a mix of miR-221- and miR-222-mimics (ADAR2+miR-221/222, red) were seeded and proliferation was monitored over days. A172 untransfected cell line (untreated, dark grey) was used as control of proliferation. *Error bars* indicate standard deviations of four independent experiments. Mean ± sd (n=4), **p<0.01 when untreated and ADAR2 plus miR-221/222 cell lines are compared to the others (ADAR2 and ADAR2 scr). (**b**) The protein lysates extracted from the same cells used in (**a**) were analysed for $p27^{Kip1}$ protein level by western blotting at 48h and 72h post transfection. (**c**) PDCD4 immunoblotting of total protein extracts from A172 ADAR2 and ADAR2 E/A cell lines and the corresponding quantitative densitometric analysis are shown. Each sample was normalized to GAPDH and compared to the ADAR2 E/A cells arbitrarily set to 1. One representative of 2 independent experiments is shown. (**d**) Representative photographs of A172 ADAR2 cells transfected with scramble mimic (scr) and miR-21 mimic (miR-21) at 0, 15 and 21 hours after scratching the surface of a cell monolayer. Only the A172 ADAR2 plus miR-21 cells show an increased motility compared to the control cells (A172 ADAR2 plus scr). No alteration of cell proliferation, in identical conditions, was reported upon miR-21 overexpression (data not shown).

Figure S7. Cell proliferation of U118 ADAR2 E/A cell line after miR-221/222 transfection. (**a**) $8x10^4$ U118 cells (untreated, dark grey), U118 ADAR2 E/A transiently transfected with either scramble mimic (200nM) (scr, light grey) or miR-221- plus miR-222-mimics (final concentration of 100nM, black, and 200nM, red) were monitored over days. *Error bars* indicate standard deviations of two independent experiments. Mean ± sd (n=2), **p*<0.05 when ADAR2 E/A plus miR-221/222 (100 and 200nM) cell lines were compared to the scramble. (**b**) Transfection efficiency of the experiment in (**a**) was tested by qRT-PCRs of the specific miRNAs. Folds of expression of the mature miR-221 (light grey, left panel) and miR-222 (middle grey, right panel) are shown. The expression levels were calculated as a relative-fold increase compared to the scramble cells arbitrarily set to 1. Each sample was normalized to RNU6B levels. Mean ± s.e.m. (n=2).

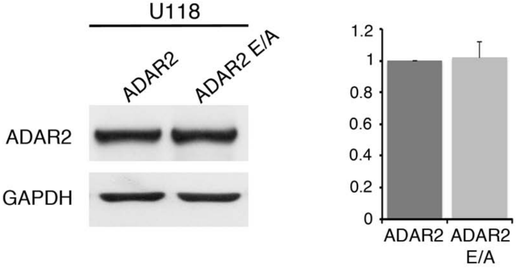
U118
ADAR2
ADAR2 E/A
ADAR2
GAPDH
1.2
1
0.8
0.6
0.4
0.2
0
ADAR2
ADAR2 E/A

Figure S1

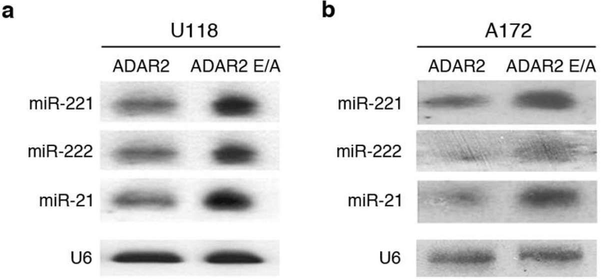
a
U118
ADAR2
ADAR2 E/A
miR-221
miR-222
miR-21
U6
b
A172
ADAR2
ADAR2 E/A
miR-221
miR-222
miR-21
U6

Figure S2

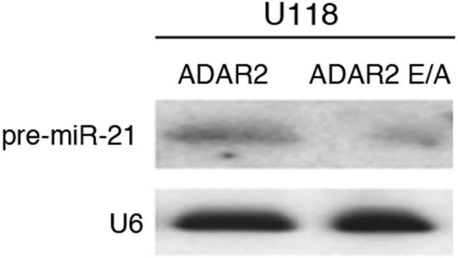

Figure S3

a

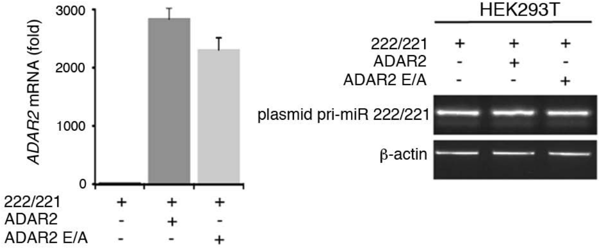
ADAR2 mRNA (fold)
3000
2000
1000
0
222/221 + + +
ADAR2 - + -
ADAR2 E/A - - +
HEK293T
222/221 + + +
ADAR2 - + -
ADAR2 E/A - - +
plasmid pri-miR 222/221
β-actin

b

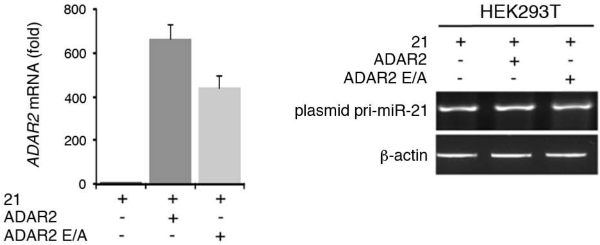
ADAR2 mRNA (fold)
800
600
400
200
0
21 + + +
ADAR2 - + -
ADAR2 E/A - - +
HEK293T
21 + + +
ADAR2 - + -
ADAR2 E/A - - +
plasmid pri-miR-21
β-actin

Figure S4

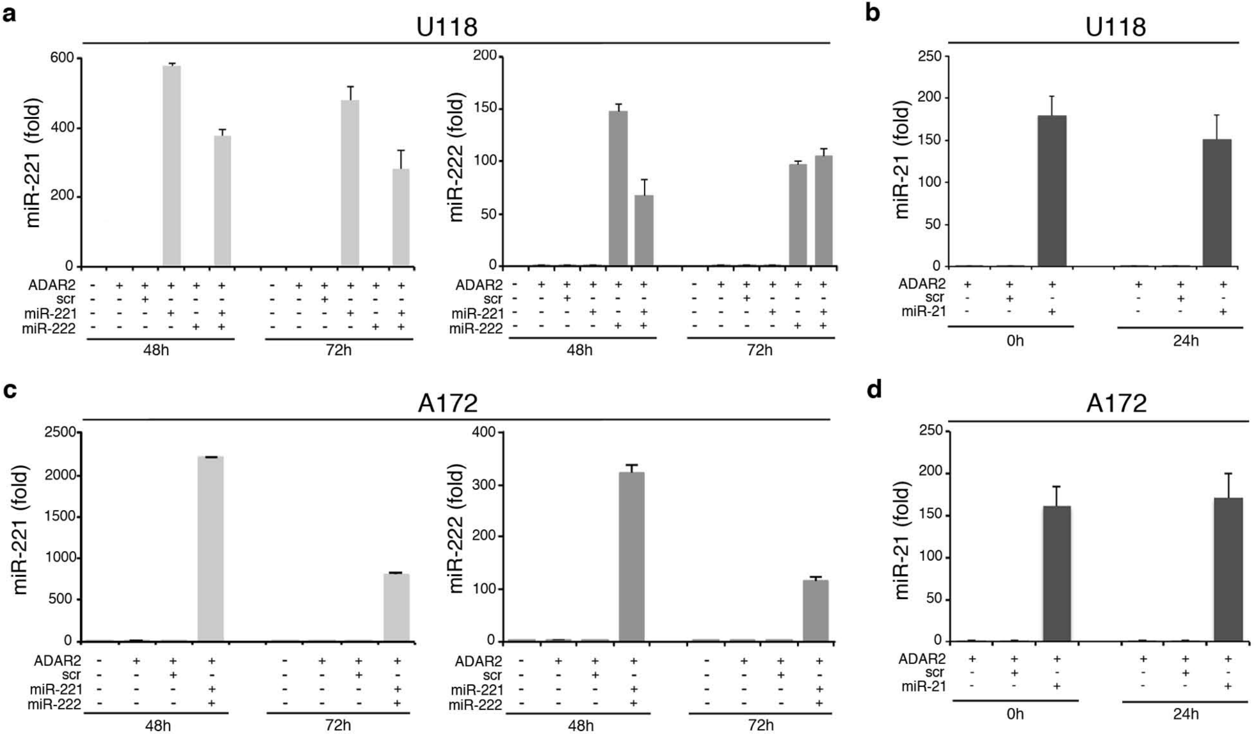
a
U118
miR-221 (fold)
miR-222 (fold)
ADAR2
scr
miR-221
miR-222
48h
72h
b
U118
miR-21 (fold)
ADAR2
scr
miR-21
0h
24h
c
A172
miR-221 (fold)
miR-222 (fold)
ADAR2
scr
miR-221
miR-222
48h
72h
d
A172
miR-21 (fold)
ADAR2
scr
miR-21
0h
24h

Figure S5

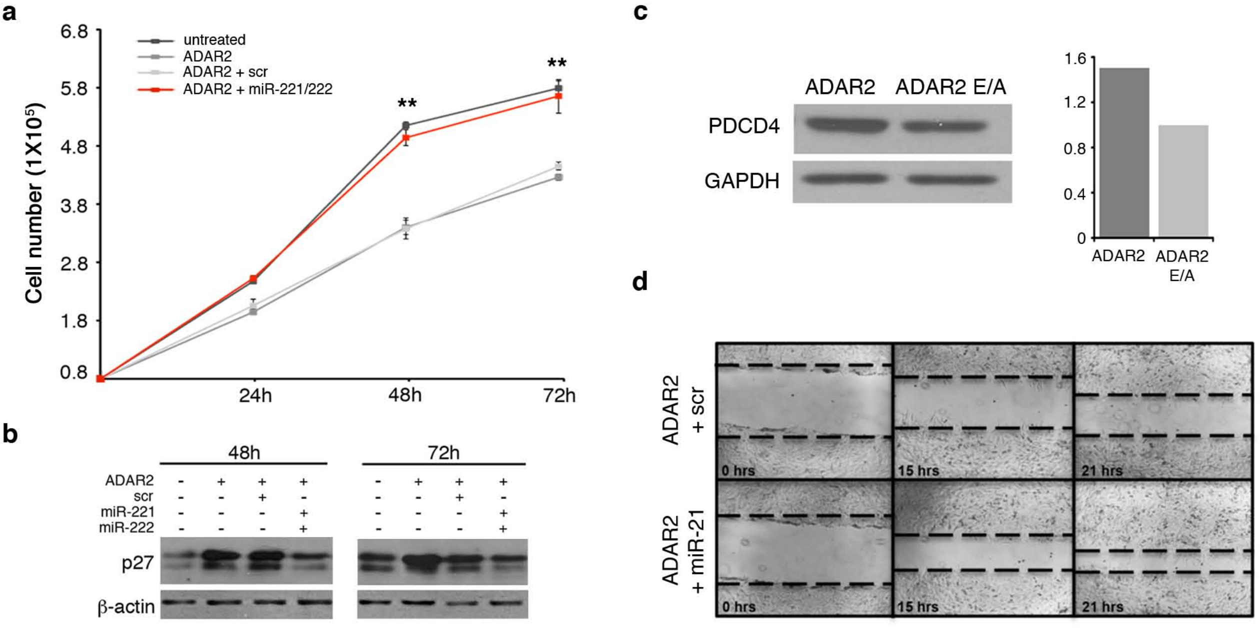

a
6.8
5.8
4.8
3.8
2.8
1.8
0.8
Cell number (1X10^5)
untreated
ADAR2
ADAR2 + scr
ADAR2 + miR-221/222
**
**
24h
48h
72h
b
48h
72h
ADAR2
scr
miR-221
miR-222
p27
β-actin
c
ADAR2
ADAR2 E/A
PDCD4
GAPDH
1.6
1.2
0.8
0.4
0
ADAR2
ADAR2 E/A
d
ADAR2 + scr
ADAR2 + miR-21
0 hrs
15 hrs
21 hrs

Figure S6

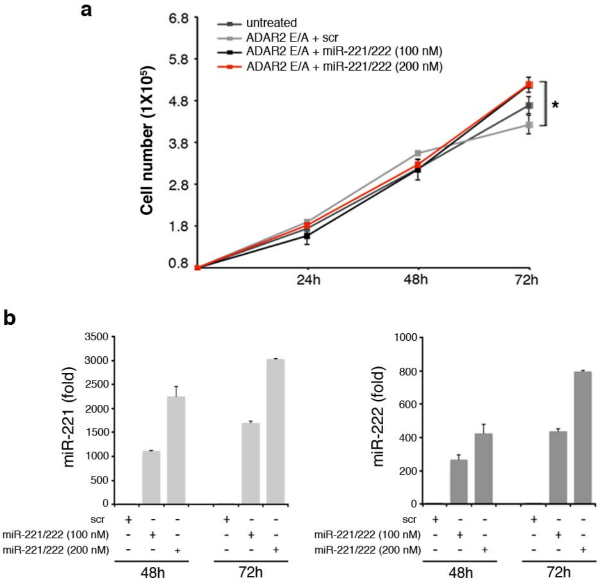
a
untreated
ADAR2 E/A + scr
ADAR2 E/A + miR-221/222 (100 nM)
ADAR2 E/A + miR-221/222 (200 nM)
Cell number (1X10⁵)
6.8
5.8
4.8
3.8
2.8
1.8
0.8
24h
48h
72h
*
b
miR-221 (fold)
3500
3000
2500
2000
1500
1000
500
0
miR-222 (fold)
1000
800
600
400
200
0
scr
miR-221/222 (100 nM)
miR-221/222 (200 nM)
48h
72h

Figure S7
